# Effects of the timing of grazing on insect diversity and insect–plant interactions in mountain grasslands

**DOI:** 10.1002/eap.70129

**Published:** 2025-11-11

**Authors:** Bernd Panassiti, Jörg Ewald, Martina Hofmann, Valeria Trivellone, Verena Styrnik, Herbert Nickel, Johann Neumayer, Katharina Pospisil, Denise Klein, Cynthia Tobisch, Sebastian König, Tobias Richter, Lisa Geres, Roland Baier, Sebastian Seibold

**Affiliations:** ^1^ Institute for Ecology and Landscape University of Applied Sciences Weihenstephan‐Triesdorf Freising Bavaria Germany; ^2^ Sustainable Agriculture and Energy Systems University of Applied Sciences Weihenstephan‐Triesdorf Freising Bavaria Germany; ^3^ Illinois Natural History Survey, Prairie Research Institute University of Illinois at Urbana‐Champaign Champaign Illinois USA; ^4^ Bavarian State Institute of Forestry Freising Bavaria Germany; ^5^ Göttingen Lower Saxony Germany; ^6^ Elixhausen Salzburg Austria; ^7^ Kierling Lower Austria Austria; ^8^ Naturpark Nagelfluhkette e.V. Immenstadt im Allgäu Bavaria Germany; ^9^ Berchtesgaden National Park Berchtesgaden Bavaria Germany; ^10^ School of Life Sciences, Ecosystem Dynamics and Forest Management Group Technical University of Munich Freising Bavaria Germany; ^11^ Faculty of Biological Sciences, Institute for Ecology, Evolution and Diversity, Department of Conservation Biology Goethe University Frankfurt Frankfurt am Main Hessen Germany; ^12^ Chair of Forest Zoology TUD Dresden University of Technology Tharandt Saxony Germany

**Keywords:** alpine grassland, Auchenorrhyncha, bees, butterflies, functional diversity, grazing management, insect diversity, insect traits, insect–plant interaction, network diversity, phylogenetic diversity, pollinator networks

## Abstract

Grazing is the common agricultural land‐use in mountain regions. It is of high socioeconomic importance but also essential for conservation as extensive mountain pastures are hotspots of biodiversity. Climate change is causing earlier growing seasons, prompting earlier livestock turnout. The effects of grazing on biodiversity, however, may differ depending on the time of the year, yet our understanding of these effects is limited. Here, we evaluate how short‐term effects of different livestock turnouts affect taxonomic, phylogenetic, and functional diversity of pollinators (wild bees and butterflies) and phytophagous insects (leafhoppers) as well as plant–insect interactions on eight mountain pastures in the northern Alps, Germany. At each pasture, we established three grazing treatments including an ungrazed control, early and late livestock turnout. We sampled wild bees and butterflies during two and leafhoppers during one growing season twice a year (summer onset and summer peak). To account for effects of grazing through changes in vegetation, we surveyed vegetation characteristics, such as the number of inflorescences and sward height. Early‐grazing plots had lower wild bee and leafhopper diversity during summer onset, but this pattern shifted later in the season after grazing had stopped. During summer peak, wild bee diversity was higher at early‐grazing plots than at late‐grazing plots and structural equation modeling indicated that this could be partly explained by a higher number of inflorescences. Phylogenetic network diversity of wild bee– and leafhopper–plant networks was higher at late than at early‐grazing plots. Our study shows that grazing in general, and also the timing of grazing, affects vegetation characteristics, insect diversity, and plant–insect interactions in mountain pastures. Effects of grazing on insect diversity were mostly positive, which supports the notion that extensive grazing is important to maintain insect diversity in mountain pastures below the timberline. Although negative effects of early livestock turnout treatments occurred, they disappeared and even turned positive later in the season. Thus, earlier livestock turnout does not appear to threaten insect diversity in mountain pastures, but further research is needed to understand long‐term effects.

## INTRODUCTION

Livestock grazing is a traditional land‐use practice in many mountain regions worldwide. Mountain pastures are of high socioeconomic importance, not only for farmers but also, due to their cultural heritage and their attractiveness, for tourism (Schirpke et al., 2016). In anthropogenic grasslands, such as mountain pastures below the tree line, grazing is needed to maintain grassland vegetation types and prevent shrub and tree encroachment (MacDonald et al., 2000). In addition, extensive mountain pastures encompass numerous protected habitats (European Union Habitats Directive 92/43/EEC, 2013) and host high levels of biodiversity, particularly of plants and insects (Ewald et al., 2025; Fontana et al., 2020; Väre et al., 2003), including many rare and threatened species. Maintaining mountain pasture systems is therefore highly relevant for both socioeconomic and ecological reasons. Climate change is strongly affecting mountain ecosystems (Broadbent et al., 2024; Steinbauer et al., 2018) and has become a major challenge for the management of mountain pastures. Low temperatures and short growing seasons are the main limiting factors for mountain species (Körner, 2021). Thus, increasing temperatures and longer growing seasons due to climate change are expected to cause shifts in plant and insect phenology (Totland, 1999; Wang et al., 2020; Zhu et al., 2021), enhance plant growth rates (Körner, 2021), and increase total insect biomass (Welti et al., 2022). On the other hand, warming‐induced drought can negatively affect plant biomass (Geange et al., 2017; Peterson & Billings, 1982), and higher temperatures lead to declines and elevational shifts of cold‐adapted species (Braziunas et al., 2024; Wessely et al., 2022). Pasture management is adapting to an earlier start of the growing season by earlier livestock turnout to make use of high‐quality forage at the beginning of the growing season and to prevent the encroachment by grasses and shrubs, which are consumed by livestock only early in the season when their palatability is higher (Ganskopp et al., 1993). Conservationists, however, are concerned that earlier turnout dates might have negative effects on plant and insect diversity arguing that when early bloomers are more heavily grazed they become more vulnerable to frosts, with cascading effects on insect communities (Pardee et al., 2018). Moreover, turnout dates are often legally fixed and thus do not allow earlier turnout.

Grazing can affect insect communities either directly or indirectly (Van Klink et al., 2015). Direct effects of grazing via trampling cause mortality of individuals and the modification of habitats, such as pollinator nesting sites (Ebeling et al., 2012; Potts et al., 2005; Sugden, 1985; Williams et al., 2010). Grazing can indirectly affect insect communities through changes in vegetation structure and diversity, soil characteristics or microclimatic conditions (Debano, 2006; Huntly, 1991; Kruess & Tscharntke, 2002b; Strong et al., 1984). Extensively grazed pastures host a higher insect diversity compared to intensively grazed ones (Kruess & Tscharntke, 2002a; Rakosy et al., 2022). However, although some insect groups can benefit from suspended grazing in the short term (e.g., higher sward height and flower abundance, Biedermann et al., 2005; Davidson et al., 2020; Sjödin et al., 2008), insect composition may change when woody encroachment leads to a shift in vegetation composition (Hussain et al., 2023; Sõber et al., 2024).

In contrast to grazing intensity, only a few studies explored the effect of the timing of grazing on biodiversity. A study conducted across 10 upland grasslands in Montana (USA), including an ungrazed control, early turnout (mid‐June), and late turnout (mid‐July) found that both early and late‐grazing treatments had similar negative effects on the abundance of most arthropods, but the impact was delayed in the late treatment (Davis et al., 2014). The authors concluded that early season grazing is less advantageous for insects emerging early in the season (Davis et al., 2014). Another study conducted at 11 lowland grasslands in Lesvos Island (Greece) found that early grazing may promote the abundance of dominant bee species that suppress less competitive ones, leading to overall higher abundances but lower species richness (Lázaro et al., 2016).

Furthermore, the abundance and species richness of small‐bodied arthropod predators, such as ants, carabids and spiders were shown to benefit from early grazing in south‐central Sweden, possibly related to microclimatic conditions (Lenoir & Lennartsson, 2010). Yet, it remains unclear if these findings on pollinators and phytophagous insects are applicable to the European Alps and how plant–insect networks at mountain pastures are affected by different turnout dates.

Here, we evaluated the effect of different livestock turnout dates on pollinator and phytophagous insect diversity as well as plant–insect networks in mountain pastures in a Biosphere Reserve in the northern European Alps. At each of eight mountain pastures, we established three grazing plots (treatments) including control (no grazing) and early and late livestock turnout. We sampled two pollinator groups, that is, wild bees (Hymenoptera: Apiformes) and butterflies (Lepidoptera: Papilionoidea), for two subsequent growing seasons (2 years) and leaf‐ and planthoppers (Hemiptera: Auchenorrhyncha), a strictly phytophagous group, for one growing season (1 year). All groups were sampled twice a year to reflect two different phenological windows (summer onset and summer peak), and the period when either the early‐grazing or late‐grazing treatment was recently grazed. These three insect groups are associated with their host plants and, therefore, respond quickly to changes in vegetation composition and structure (Geppert et al., 2021; Lengyel et al., 2016; Menéndez et al., 2007; Schaffers et al., 2008). In addition to the insect sampling, we recorded the number of inflorescences and sward height—two vegetation parameters relevant to insects—responding to grazing in short term (Davis et al., 2014; Kruess & Tscharntke, 2002a; Lázaro et al., 2016; Lennartsson et al., 2012; Vulliamy et al., 2006), and we recorded the plant species on which pollinators were observed. We evaluated effects of grazing and different turnout dates using mixed generalized models and we applied structural equation models (SEMs) to assess whether grazing effects are mediated by vegetation characteristics. Since the effects of grazing and livestock turnout on plant–insect interactions in mountain pastures are not well understood, we constructed networks of pollinator–plant and phytophage–plant interactions and estimated their taxonomic, phylogenetic, and functional network diversity (TD, PD, and FD, respectively). In particular, we addressed the following research questions: (Q1) How do grazing in general and different livestock turnout dates in particular affect the TD, PD, and FD of pollinators and phytophagous insects during summer onset and during summer peak? (Q2) Is the effect of different livestock turnout dates on pollinators and phytophagous insects direct or indirectly mediated via vegetation characteristics? (Q3) Do different livestock turnout dates affect the TD, PD, and FD of plant–insect pollinator and phytophagous networks?

## MATERIALS AND METHODS

### Study area and sampling design

**FIGURE 1:**
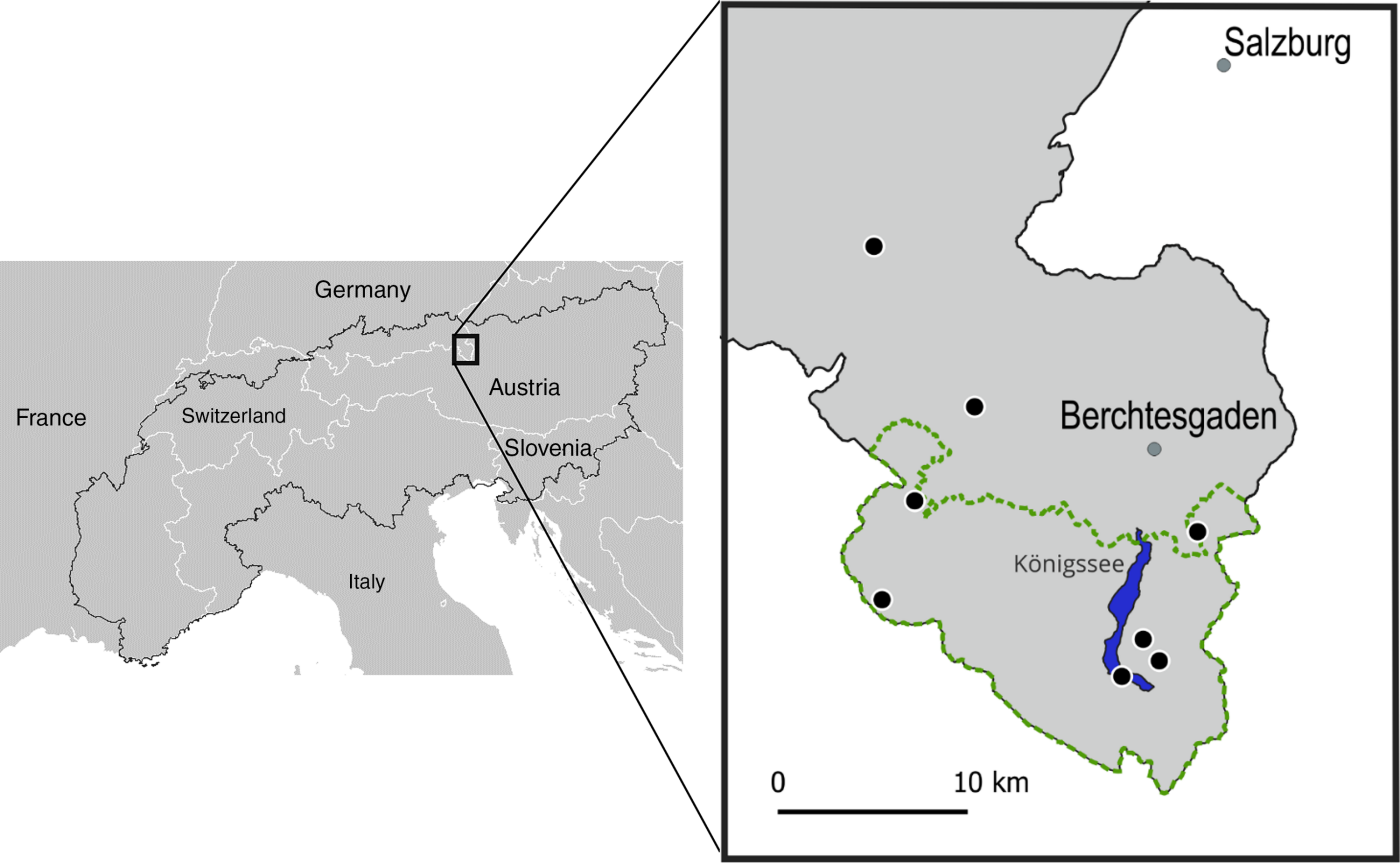
Study area in the Bavarian Alps of southern Germany. (a) Boundary of European Alps indicated by the black line. (b) Study area with eight mountain pastures indicated by black dots. Greenish dotted line outlines the border of the Berchtesgaden National Park (*Source*: ESDAC, 2024).

Our study region is part of the Northern Limestone Alps in southern Germany. The study sites included eight mountain pastures located in the UNESCO Biosphere Reserve Berchtesgadener Land with six of them also located within Berchtesgaden National Park (Figure 1) between 600 and 1740 m above sea level (asl) The land of the mountain pastures belongs to the State of Bavaria and local farmers are allowed to use it for pasturing, often with a century‐long tradition. The bedrock of the study sites is characterized by limestone, dolomite or radiolarian rock with a mineral soil pH_KCl_ ranging from 3.42 to 6.96 (Appendix S1: Table S1). The pasture vegetation is dominated by the grasses (Poaceae) *Nardus stricta* on acidic, *Dactylis glomerata* on intermediate and *Sesleria caerulea* as well as the ericoid dwarf shrub *Erica carnea* on neutral to alkaline soils (Ewald et al., 2025). With increasing elevation, mean annual temperature decreases from 7 to −2°C and mean annual precipitation increases from 1500 to 2800 mm (Nationalpark Berchtesgaden, 2023). Since 1880, the mean annual temperature has increased by 1.8°C in the Alps (Auer et al., 2007; Begert & Frei, 2018), and between 1958 and 2019, the onset of the growing season in the Swiss Alps occurred 2.8 ± 1.3 days earlier per decade (Vorkauf et al., 2021).

On each of the eight mountain pastures, we established three grazing treatments before the start of the grazing season in 2021, resulting in a total of 24 plots. Using electric fences, each pasture was divided into two halves with similar topography and vegetation and we randomly assigned one half to early and the other to late turnout treatment. The size of the treatment plots averaged ~8 ha (±~4 ha, see Appendix S1: Table S1). In addition, an ungrazed fenced area of approximately 900 m^2^ within the grazed area and in between the treatment plots was set up at each pasture as an ungrazed control. The livestock turnout dates in the early‐turnout treatment were selected based on the vegetation development in each year (2021 and 2022) with grazing starting shortly after the snow melt as soon as the vegetation sustained livestock. Depending on the elevation of the pasture, early‐turnout dates varied between early May and mid‐June (Appendix S1: Table S2). Late‐turnout dates in our study corresponded to turnout dates of the past decades which are legally fixed for each pasture in the regulations of the right of use. Late‐turnout dates varied with elevation between the end of May and the end of July. These dates have not changed for decades and presumably reflect the vegetation phenology before climate change. Late‐turnout dates were on average 3 weeks later than early‐turnout dates of the same pasture. On each pasture rotational grazing was carried out; that is, livestock was first moved to the early‐turnout treatment and then moved to the late‐turnout treatment. After the vegetation on the late‐turnout plot was grazed, livestock was either moved back to the early turnout and then to the late‐turnout treatment again or livestock was allowed to roam freely on the entire pasture (see Appendix S1: Table S2 for turnout and turnover dates). All pastures were grazed exclusively by cattle including dairy cows and heifers with a carrying capacity of on average of 111 standard livestock units (one standard unit reflects 500‐kg livestock) grazing days per hectare and grazing season (Appendix S1: Table S1).

### Data collection

#### Insect sampling

We surveyed pollinators in 2021 and 2022 and phytophagous insects in 2021. Insect sampling was carried out twice a year reflecting different phenological windows and vegetation states. The first sampling occurred during early summer, usually at the end or shortly after the period of the early‐turnout treatment (hereafter summer onset) and the second sampling occurred during mid or late summer toward the end or shortly after the period of the late‐turnout treatment (hereafter summer peak). An overview of grazing and sampling times is provided in Appendix S1: Table S2. Due to logistical constraints such as weather conditions, in a few instances, insect sampling was conducted while cattle were still present on the plots.

We focused on two pollinator groups: butterflies (Lepidoptera: Papilionoidea, including Zygaenid moths) and wild bees (Hymenoptera: Apiformes). For pollinators, we randomly selected a 25 m × 29 m subplot within each treatment plot in which butterflies and bumblebees were recorded along six adjacent 25‐m transects with an observation width of 4 m, thus covering a total of 600‐m^2^ per plot. In addition, all other wild bees were collected along two 25‐m transects with a width of 1 m located at the plot center, amounting to a total of 50‐m^2^ per plot. We recorded the presence, abundance, and interactions of pollinators with flowers, including the specific plant species and whether the pollinators were foraging. Identification was carried out in the field using a sweep net. Individuals were captured for visual inspection and released or collected and transferred to the laboratory for identification if identification in the field was not possible. We used the standard identification keys for butterflies (Baudraz et al., 2020; Stettmer et al., 2022) and wild bees (Amiet, 1996; Amiet et al., 1999, 2001; Scheuchl, 1996; Schmid‐Egger & Scheuchl, 1997). Pollinators were recorded during dry weather conditions, with a maximum of 75% cloud cover and low wind speeds. Due to logistical reasons, pollinator surveys were not carried out in site 1 (Höllenbachalm) in 2022, in site 4 (Mittereisalm) during summer onset in 2022, in site 7 (Gotzenalm) during summer peak in 2022 and in site 8 (Regenalm) during summer onset in 2021.

As phytophagous insects, we selected leaf‐ and planthoppers (Hemiptera: Auchenorrhyncha, hereafter referred to as leafhoppers). Leafhoppers were sampled along a 50‐m transect at each plot using a sweep net and D‐Vac suction sampler with a total of 150 sweeps and 75 suctions, respectively, over the two sampling times. We recorded presence and abundance. Taxonomy follows Nickel and Remane (2002) and Nickel et al. (2016). A planthopper–plant network matrix was scored for the species known to be strictly associated with one host plant species (defined as monophagous 1st grade − m1, Nickel & Remane, 2002). We recorded the presence of an interaction if an m1‐planthopper and the corresponding host plant co‐occurred in the same plot.

### Vegetation parameters

As an indicator for flower presence and abundance, we recorded the number of inflorescences (pollination units sensu Faegri & Van der Pijl, 1966) within the two pollinator transects during both pollinator surveys. As vegetation height is an important parameter for phytophagous insects (Biedermann et al., 2005), we also measured sward height using a pasture meter (Jenquip EC20 Pasture Meter, https://pasturemeters.co.uk/product/ec20-pasture-meter/). The pasture meter measures the compressed sward height by means of a rising plate (Castle, 1976). Sward height was recorded along a transect in each treatment using 100 sample locations (measurements) in early and late treatments to account for grazing activity. Because our aim was to characterize the vegetation structure representative of the entire pasture, and to maintain comparability across sites, we excluded sampling points that overrepresented extensive areas. As a result, the final number of valid points for pasture A004 at those dates was lower (early treatment: 15 in summer onset and 46 in summer peak, 30 control). To correlate vegetation height and species richness of leafhoppers, we used the closest measurement date of sward height before the leafhopper survey and averaged over all measurements. In case of an additional measurement date shortly after the leafhopper survey, we averaged sward height measurements of both dates.

We also recorded and analyzed plant species richness and plant cover, although these variables were not expected to change in response to grazing treatments within the 2 years of our study. As expected, the differences among treatments were mostly negligible, and thus, methods and corresponding findings are included in Appendix S1. The methods are described in the section titled “Vegetation Survey” and the results are presented in Appendix S1: Figure S1.

### Phylogenetic trees

The barcode sequences for all insect species were obtained from the German Barcode of Life database (Geiger et al., 2016; Wägele et al., 2023) and the National Center for Biotechnology Information (NCBI) database (Federhen, 2012). The final molecular datasets included 35, 59, and 97 taxa for wild bees, butterflies, and leafhoppers, respectively. Sequences of each dataset were edited and aligned using the Muscle algorithm with default settings in MEGA 7.0 (Edgar, 2004; Kumar et al., 2018). The aligned sequences were analyzed in PartitionFinder (v. 2.1.1), using the corrected Akaike information criterion (AIC_c_) selection criteria to identify appropriate substitution models for tree construction (Lanfear et al., 2012, 2017). The trees were constructed using the general time reversible (GTR) model with gamma‐distributed rate variation and invariable sites (GTR + G + I) that were associated with the lower AIC_c_ for each dataset. Phylogenetic trees were constructed using the Maximum Likelihood approach in RAxML (Stamatakis, 2014) using raxmlGUI 2.0 (Edler et al., 2021).

### Functional traits

Response of species' populations to environmental changes varies depending on habitat preferences, ecological tolerance, and dispersal patterns. Studies have shown that pollination efficiency relates to proboscis length and the length of corolla tubes (Inouye, 1980; Szigeti et al., 2020), and thus structures plant–pollinator networks (Corbet, 2000). Moreover, species with a narrow host repertoire may have specific habitat requirements such as meadows with low nitrogen inputs (Habel et al., 2022; Nickel & Achtziger, 2005). To account for those physiologically, morphologically and ecologically constrained plant–insect associations, we compiled a set of ecological and life‐history traits for pollinators and leafhoppers. In total, we selected 12 traits (feeding niche, wing length, migration propensity, distribution index, population density, number of eggs, voltinism, egg maturation, hibernation stage, flight period, adult feeding, proboscis length) for butterflies (Higgins & Riley, 1978; Settele et al., 1999, 2009), eight (proboscis length, life strategy, body size, voltinism, flight period, social behavior, nest type, nest location) for wild bees (Westrich, 2019), and seven (life strategy, body length, pasture indicator, diet width, aridity range, overwintering, voltinism) for leafhoppers (Biedermann & Niedringhaus, 2009; Nickel, 2003). Moreover, we selected six traits associated with morphological and qualitative features increasing pollinator attraction (flower size, display size, UV reflectance periphery, UV reflectance patterns, nectar tube depth, flower color) for pollinator‐visited plants and 13 traits (stem specific density, nitrogen (N) fixation capacity, leaf N content per dry mass, leaf phosphorus (P) content per dry mass, N content per leaf area, leaf dry mass, N/P ratio, leaf length, carbon (C)/N ratio, plant height, leaf area, leaf area per dry mass, leaf cell wall mass per dry mass) for host plants of monophagous leafhoppers (TRY database, Kattge et al., 2020). Appendix S1: Table S3 provides a complete list of all traits.

### Statistical analyses

We applied a coverage‐based (i.e., sample completeness) rarefaction and extrapolation approach to calculate TD, PD, FD, respectively (Hill number *q* = 0) using the *estimate3D* function from the R package iNEXT.3D (v. 1.0.1, Chao et al., 2021). We used abundance data and standardized per plot. We fixed sample coverage for all three groups to 0.7. Diversity estimates were used to answer research questions Q1 and Q2 (see below). To answer research question Q3, we estimated coverage‐based network diversity estimates of plant–insect interactions for each insect group using the functions *iNEXT.link* and *estimateD.link* (v. 1.0.1, Chiu et al., 2023), and the default 30 bootstrap replications to assess sampling uncertainty. Estimates were calculated for taxonomic, phylogenetic, and functional network diversity (TDnet, PDnet, and FDnet, respectively) using species observations, phylogenetic trees and dissimilarity matrices. Dissimilarity matrices using functional traits were calculated using Gower's distance with the function *gower.dist* from the R package StatMatch (v.1.41, D'Orazio, 2022).

We fitted generalized linear mixed models (GLMMs) and SEMs to explore the effects of livestock turnout dates on standardized diversity estimates and vegetation parameters. For each insect group (wild bees, butterflies, leafhoppers), we used the estimates of TD, PD, and FD (diversity type) as response variables. To address research question Q1, we fitted two different GLMM models for each insect group and diversity type. One model included an interaction of turnout dates (control, early, late) and survey period (two levels; 1: summer onset and 2: summer peak) as explanatory variables, and one model included an interaction term of a vegetation variable and survey period. We used the number of inflorescences in wild bee and butterfly models, and sward height in leafhopper models. To address research question Q2, we used SEMs that hierarchically fit vegetation as a function of grazing treatments and insect diversities as a function of vegetation and grazing treatments. All models included sampling year (except for leafhoppers) and elevation as fixed factors and the site as a random effect (i.e., allowing the intercept to vary for each site) to account for the nested design and repeated sampling.

GLMM and SEM were fitted as regression models using a Gaussian error distribution. The models were built via a full Bayesian inference using the “brm” function in the *brms* package (v. 2.20.4, Bürkner, 2017). This function uses the Stan platform (v. 2.32.3, Stan Development Team, 2023) that implements the No‐U‐Turn Sampler, an extension to the Hamiltonian Monte Carlo algorithm that eliminates the need to set a number of leapfrog steps (Hoffman & Gelman, 2014). We chose 5000 iteration steps and four chains. The four chains were considered to have converged when the R‐hat convergence diagnostic was <1.05 (Gelman, 2014). Moreover, we controlled the sampler behavior to avoid divergent transitions after warmup and hence biased posterior draws. Therefore, we slowed the sampling speed using the argument “adapt_delta” set to 0.9999 and increased the tree depth to be evaluated by setting the argument “max treedepth” = 15. Fixed effects priors were normally distributed and centered around zero. We set the fixed effect prior to be mildly informative (SD = 10) which applies a shrinkage comparable to a ridge regression. For random effects priors default settings were used, that is, half student‐t priors with 3 degrees of freedom and a scale parameter of 2.5.

The residual distribution was inspected using the *DHARMa* package (v. 0.4.6, Hartig, 2023), and for none of the models serious residual problems were identified. Across all fitted GLMM and SEM, the Bayesian *R* ^2^ ranged from 0.25 to 0.67 and 0.26 to 0.83. We used Pareto smoothed importance‐sampling to check the reliability of the estimates (Vehtari et al., 2017). The Bayesian version of *R* ^2^ conditioning on fixed as well as random effects was calculated using the function “bayes_R” from the *brms* package (Gelman et al., 2019). As we were interested in the effect of grazing (compared to control with no grazing) but also in the difference between early and late grazing, we applied planned contrasts using the functions “emmeans” from the *emmeans* package (v. 1.8.9, Lenth, 2023) and “contrasts” (*stats* base package). We conducted all statistical analyses using the software R (v. 4.3.1, R Core Team, 2023).

## RESULTS

In total, we recorded 448, 442, and 7657 individuals and 27, 56, and 95 species of wild bees, butterflies, and leafhoppers, respectively.

### (Q1) effects of livestock turnout dates on wild bees, butterflies, and leafhoppers

**FIGURE 2:**
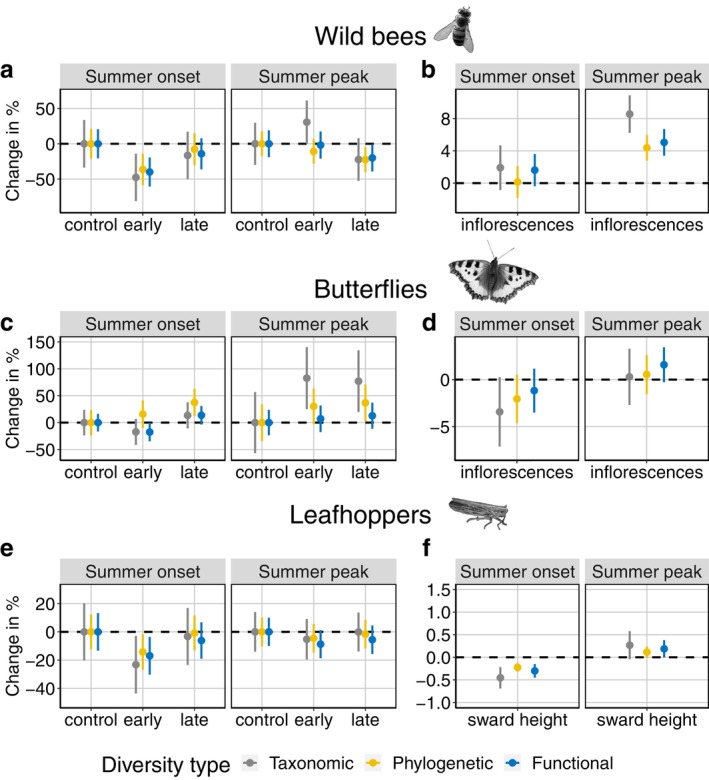
Percent change in diversity of wildbees, butterflies, and leafhoppers as a function of early and late livestock turnout treatments compared to control with absence of grazing (a, c, e) and vegetation parameters (b, d, f). Percent change is indicated by points, lines show corresponding SEs. Taxonomic, phylogenetic, or functional diversity, highlighted in gray, golden yellow, and blue, respectively, are calculated for Hill number *q* = 0 at standardized sample coverage of 0.7. The change in percentage in wild bee, butterfly, or leafhopper species is quantified as a result of 100 additional inflorescences (flowers); or an increase of sward height by 1 cm (sward height). Original wild bee and leafhopper images provided by Pexels were modified and used under the Pexels License. The original butterfly image was provided by Katharina Pospisil and subsequently modified.

Wild bee TD, PD, and FD were on average 28%, 25%, and 19% lower in the first period (summer onset) compared to the second period (summer peak; Figure 2a). During summer onset, we found a small insignificant difference in wild bee diversity (TD, PD, FD) among early and late turnout treatments. Wild bee diversity was lower for the early turnout treatment compared to the control, whereas the late turnout treatment was similar to the control. During summer peak, wild bee TD differed between the early and late turnout treatment (posterior probability = 0.94) with TD being 31% higher in the early turnout treatment compared to the control, but similar for late turnout and control. PD and FD were similar to the control for both turnout treatments during summer peak.

Butterfly TD, PD, and FD were on average 34%, 21%, and 24% higher during summer onset compared to summer peak (Figure 2c). During summer onset, butterfly TD, PD, and FD did not significantly differ among early and late treatments (Figure 2c). During summer peak, butterfly diversity (TD, PD, FD) was on average ~40% higher in both grazed treatments compared to the control (Figure 2c), but early and late treatments did not differ significantly either.

Leafhopper TD, PD and FD were on average 35%, 23%, and 26% lower during summer onset compared to summer peak (Figure 2e). During summer onset, we found a small but nonsignificant difference for all diversity estimates (TD, PD, FD) between the early and late turnout treatments, on average ~15% lower in early treatments. Leafhopper diversity was lower for the early turnout treatment compared to the control, whereas the late turnout treatment was similar to the control. During summer peak, leafhopper diversity (TD, PD, FD) differed slightly between early and late treatments, on average ~4% lower in early treatments. Leafhopper diversity in early turnout treatments and late turnout treatments was similar to the control.

The number of counted inflorescences was 8% lower during summer onset compared to summer peak (Appendix S1: Figure S1). Even though not statistically significant, we found that the number of inflorescences was higher in the early treatment compared to the late one during summer onset and summer peak. Compared to the control, both grazed treatments (early and late) also had a lower number of inflorescences in both survey periods. However, we only found a significant difference between the control and late turnout treatments during summer peak, with on average 356 fewer inflorescences (95% CI: −698 to −13.8, *p* < 0.05). Sward height was 14% higher during summer onset compared to summer peak (Appendix S1: Figure S1). Sward height was always lower in grazed treatments compared to control, but only the late turnout treatment had significantly lower sward height compared to the control, with a mean difference of −23.9 (95% CI: −44.8 to −3.13, *p* < 0.05).

Wild bee diversity (TD, PD, and FD) increased with the number of inflorescences during the summer peak, but not during the summer onset (Figure 2b). We found no significant effect of the number of inflorescences on butterfly diversity (Figure 2d). TD, PD, and FD of leafhoppers decreased with sward height during the summer onset, but not during the summer peak (Figure 2f).

### (Q2) direct and indirect effects of livestock turnout dates on wild bees, butterflies, leafhoppers

**FIGURE 3:**
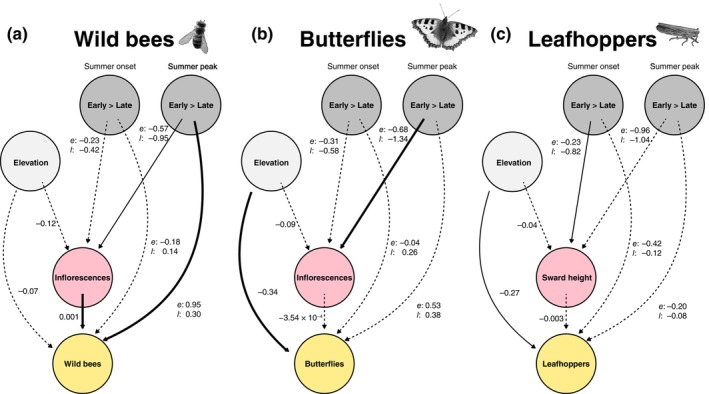
Structural equation models (SEMs) showing direct and indirect effects on the taxonomic species richness‐based diversity (TD) of (a) wildbees, (b) butterflies, and (c) leafhoppers. Insects were collected in two sampling periods (summer onset and summer peak). Treatments included early and late livestock turnout (early vs. late). Indirect effects of elevation and treatment via the number of inflorescences were tested for wild bees and butterflies and via sward height for leafhoppers. Numbers next to arrows indicate standardized path coefficients (*e* = early turnout, *l* = late turnout). Results of hypothesis tests are indicated by different arrow types. We tested whether (1) the coefficients of early turnout are greater than those of late turnout, (2) the elevation coefficient is different from zero, (3) the coefficient of the number of inflorescences is different from zero, and (4) the sward height coefficient is different from zero. Posterior probabilities of hypothesis tests with *p* ≥ 0.95 are indicated by a bold solid arrow, still notable effects (0.8 < *p* < 0.95) by a solid arrow line, and less noteworthy effects (*p* ≤ 0.8) by a dashed arrow line. Original wild bee and leafhopper images provided by Pexels were modified and used under the Pexels License. The original butterfly image was provided by Katharina Pospisil and subsequently modified.

The SEM revealed a significant direct link between livestock turnout dates and wild bee diversity (TD) with higher diversity in early than late turnout treatments during summer peak (Figure 3a, Appendix S1: Table S8). Although not statistically significant, we found more inflorescences on early than late turnout plots, particularly during summer peak. Inflorescence number positively affected wild bee diversity. This effect was small but significant for wild bee TD, PD, and FD (Figure 3a, Appendix S1: Figures S3A and S4A).

We found neither a direct link between turnout dates and butterfly diversity (TD, PD, FD) nor an indirect link via the number of inflorescences, since butterfly diversity was not associated with the number of inflorescences (Figure 3b, Appendix S1: Figures S3B and S4B). Butterfly diversity significantly decreased with elevation.

We found no direct link between turnout dates and leafhopper diversity (TD, PD, FD) nor an indirect link via sward height (Figure 3c, Appendix S1: Figures S3C and S4C). In contrast to the direct effects of sward height captured by a GLMM (Figure 2f), the SEM in Figure 3c shows that sward height—indirectly affected by management practices—was not associated with leafhopper diversity.

### (Q3) Effect of turnout dates on plant–insect networks

**FIGURE 4:**
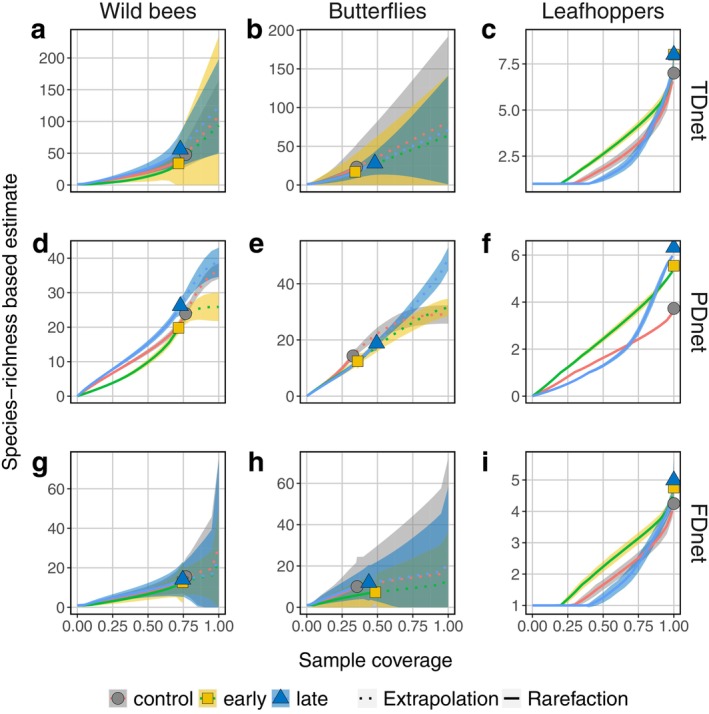
Coverage‐based rarefaction and extrapolation (*R*/*E*) curves to estimate taxonomic, phylogenetic, and functional network diversities (TDnet, PDnet, FDnet; Hill number *q* = 0) for wild bees, butterflies, and leafhoppers. For leafhoppers we used the abundance of monophagous leafhoppers and the presence of associated hostplants. Original wild bee and leafhopper images provided by Pexels were modified and used under the Pexels License. The original butterfly image was provided by Katharina Pospisil and subsequently modified.

PDnet of the plant–wild bee network was significantly higher (indicated by nonoverlapping CIs) in plots of the late treatment compared to the ones of early treatment and control (Figure 4d). For the plant–butterfly network, we found that PDnet was higher in the late treatment than in the early treatment and control when sample coverage exceeded 0.75 (Figure 4e). TDnet and functional network diversity (FDnet) did not differ significantly between turnout treatments for wild bee and butterfly networks (Figure 4a,b,g,h). For monophagous leafhoppers and associated host plants, control plots had a lower TDnet, PDnet, and FDnet than both grazed treatments. For TDnet and FDnet, network diversity was similar for both grazed treatments, but the late‐turnout treatment had a higher PDnet than the early one.

## DISCUSSION

We here investigated the short‐term effects of grazing and its timing on insect diversity, vegetation characteristics and insect–plant interactions in mountain pastures in the European Alps. Insect diversity differed between grazed and ungrazed control plots, but effects were not consistent across species groups, diversity metrics, livestock turnout dates and seasons. We observed positive effects of grazing, such as for butterfly TD during the summer peak in plots with early and late turnout dates, as well as negative effects of grazing, such as for wild bee TD on plots with early turnout during the summer onset. Differences in insect diversity between early and late turnout dates occurred for wild bee TD during the summer peak with a higher diversity at plots with early turnout dates. Livestock turnout dates affected the number of inflorescences and sward height. SEMs revealed that changes in vegetation mediated effects of turnout dates for wild bees, yet also significant direct links between turnout dates and insect diversity were observed for wild bees. Phylogenetic network diversity of wild bee–plant networks and leafhopper–plant networks was higher in late than in early livestock turnout treatments.

### Early grazing increases wild bee species richness during summer peak

Comparing ungrazed control plots to grazed areas on the same mountain pastures, we found that the number of flowers and sward height was generally lower on grazed plots. Grazing tended to have negative effects on the TD, PD, and FD of wild bees and leafhoppers during summer onset. At this time, only the early but not the late turnout treatments were grazed which likely explains why there was no significant difference between ungrazed and late‐turnout plots during summer onset. Interestingly, early grazing increased wild bee species richness (TD) during summer peak. In contrast, butterflies were generally more diverse (TD) at grazed compared to ungrazed plots during summer peak.

Previous studies have shown that flower visitors, such as wild bees and butterflies, are closely linked to the availability of food resources, such as nectar and pollen (Potts et al., 2003, 2009; Ravetto Enri et al., 2017). Our results regarding wild bees correspond to this finding since wild bee TD, PD, and FD increased with increasing numbers of inflorescences, especially during the summer peak. However, and contrary to our expectations, the number of inflorescences did not affect butterfly diversity. Similar results were obtained from SEM (see next paragraph). Although leafhoppers generally benefit from open habitats maintained by grazing, many species respond negatively to increased grazing pressure causing a reduction of vegetation height and structure (Biedermann et al., 2005). Lower vegetation height, however, did not explain lower leafhopper diversity in grazed compared to ungrazed plots during summer onset, since sward height was negatively associated with leafhopper diversity during this season. We can only speculate that microclimate or other vegetation characteristics such as grass biomass (Everwand et al., 2014) or the structural complexity of vegetation are responsible for this pattern.

### Effect of turnout dates on wild bee but not butterfly diversity is partly mediated by the number of inflorescences

Previous studies reported inconclusive effects of grazing times on arthropod biodiversity as early and late‐grazing treatments were found to have similar negative effects on the abundance of grassland arthropods (Davis et al., 2014), whereas early grazing was linked to lower bee species richness and higher bee abundance, mainly by promoting dominant species (Lázaro et al., 2016). We observed differences between early and late livestock turnout for wild bee diversity, but they differed between seasons. During summer onset, bee diversity tended to be lower in the early turnout treatment which had been grazed shortly before bee sampling. In contrast, during summer peak bee diversity was clearly higher in the early than in the late turnout treatment. Structural equation modeling indicated that the effect during summer peak can be partly explained by a reduced number of inflorescences at late‐turnout plots which had been grazed recently before bee sampling. At early‐turnout plots, plants had regrown and inflorescences were not reduced by grazing. A significant direct link between turnout treatment and bee diversity during summer peak, however, indicates other differences (such as species richness or cover of flowering plants) between turnout treatments affected wild bee diversity besides floral resources.

Although the early treatment had a higher number of inflorescences in both seasons, we found no indirect pathway from the early treatment to butterfly diversity via the number of inflorescences. We used the number of inflorescences as an indicator of pollinator units (sensu Faegri & Van der Pijl, 1966); however, other factors that were not included in our analyses may have also affected butterfly species richness. For example, Kerner et al. (2023) showed that management was a main driver of host plant species richness and flower cover; however, only host plant species richness affected butterfly species richness. Moreover, our SEM revealed a strong inverse relationship between elevation and butterfly species richness which is in line with a previous study from the same study area (Leingärtner et al., 2014). However, depending on the considered elevational gradient, a hump‐shaped pattern of butterfly species richness can also occur (Kerner et al., 2023).

Most leafhoppers respond positively to an increased sward height, as it enhances the structural complexity of the vegetation (Biedermann et al., 2005; Everwand et al., 2014; Kőrösi et al., 2012). Sward height was higher in the early treatment during summer onset. However, we found no indirect link of management via sward height and leafhopper species richness. This could be because sward height measurement dates did—for logistic reasons—not perfectly match leafhopper sampling dates. As discussed in the previous section, other not measured vegetation characteristics may better link between treatments and leafhopper diversity.

### Grazing and livestock turnout dates influence plant–insect networks

In addition to the indirect effects of livestock turnout dates on wild bee diversity via the number of inflorescences, our analyses of network diversity indicate that grazing in general as well as livestock turnout treatments affect plant–insect interactions. We found higher network diversity of leafhopper–plant networks in grazed compared to control plots and higher network diversity of wild bee–plant networks and leafhopper–plant networks in late compared to early livestock turnout treatments. However, the differences between early and late turnout treatments occurred only for phylogenetic network diversity for both taxa. Higher network diversity of leafhopper–plant networks in grazed plots may be due to higher grassland productivity (Milchunas & Lauenroth, 1993). Higher PDnet for late turnout treatments suggests that the late treatment involves interactions of more distantly related taxa than in the early treatment. For wild bees, this could be due to species, such as *Bombus monticola* and *B. pratorum* which occurred only in late‐treatment plots and which are polylectic. Polylectic pollinators can collect pollen from a wide variety of unrelated plant species, unlike oligolectic species with a narrow, specialized preference for pollen sources, typically to a single family or genus of flowering plants. The only bee species exclusively occurring in the early treatment—*Chelostoma florisomne*—was an oligolectic species.

Beyond that, we can only speculate about the reasons for this observation as different community assembly processes may have played a role in affecting the PDnet (Emerson & Gillespie, 2008; E‐Vojtkó et al., 2023; Wang et al., 2019), which were not part of our analyses.

### Study limitations

We observed significant effects of grazing and livestock turnout dates on diversity metrics of some insect taxa, but no significant effects on others, such as butterflies. The lack of statistically significant effects in our study should, however, not be considered as proof that grazing treatments have no effects on these taxa and metrics, since several factors could have limited our ability to detect significant effects. First, the timing of insect sampling had to consider phenology (e.g., not too early to avoid having only non‐adults which cannot be identified) and weather and therefore, could not always be conducted right after a grazing treatment has ended. At the same time, the timing of the grazing had to consider the state of the pasture and the forage availability, so it was not possible to, for example, extend a grazing period to wait for better weather suitable for insect sampling. Next, site conditions with respect to soil conditions and microclimate are highly variable in mountain regions even at small spatial scales (Pittarello et al., 2020), which affects species communities and diversity (Vonlanthen et al., 2006). To account for this, we created treatment areas within the same pasture, assuring that site conditions were similar across all three treatments of each pasture and included pasture ID as a random factor in our analyses. However, site conditions and plant communities differed across our eight replicated pastures (Appendix S1: Table S1) and further research is needed to assess whether effects of livestock turnout dates differ between sites, for example, between calcareous and siliceous bedrock.

While creating grazing treatments in close proximity within the same pasture was important to reduce variation that was not associated with the treatments, it could have weakened the effects of livestock turnout treatments since mobile insect species could have moved between grazing plots and concentrated in patches of preferred vegetation.

The main focus of this study was short‐term effects of different grazing regimes on insects and plant–insect interactions. Changes in plant community composition and diversity are expected only over longer time periods (Kruess & Tscharntke, 2002a), but many plant‐related drivers of insect diversity are affected within a short time through changes in grazing management, as shown for flowers (Lázaro et al., 2016; Lennartsson et al., 2012; Vulliamy et al., 2006) and vegetation height (Davis et al., 2014; Kruess & Tscharntke, 2002a). Insects were also shown to quickly respond to changes in vegetation structure and management (Helbing et al., 2023; Kruess & Tscharntke, 2002a). Nevertheless, longer term studies of effects of grazing times in mountain pastures are needed to address long‐term changes in plant communities and their effects on insect communities. Finally, our study was conducted within a protected area, where the plant and insect diversity might be higher than in other alpine pastures (e.g., for vascular plant diversity compare fig. 3 of Biurrun et al. (2021) and Appendix S1: Figure S1). Overall, our results should therefore be considered a conservative estimate of short‐term effects of grazing and turnout dates on insect communities in mountain pastures.

## CONCLUSIONS

Climate change is not only leading to changes in biodiversity in mountain ecosystems (Vitasse et al., 2021), but also poses challenges to land‐use practices. Farmers call for an adjustment of grazing times to an earlier start of the growing season to maintain productivity and prevent shrub encroachment, but conservationists are concerned that earlier grazing may negatively affect plant and insect communities (Pardee et al., 2018). We indeed observed lower wild bee diversity in early‐turnout plots compared to control plots during summer onset, but a higher wild bee diversity during summer peak, when the vegetation had regrown after grazing had stopped in the early‐grazing plots. Early‐grazing treatments held significantly higher wild bee diversity than late‐grazing plots during summer peak. Moreover, phylogenetic network diversity of wild bee–plant networks and leafhopper–plant networks was higher in late than in early livestock turnout treatments. Our results thus do not support concerns that early livestock turnout has negative effects on insect diversity.

Since our study evaluated short‐term effects and rotational grazing systems, we cannot rule out that negative effects may occur over several years of early livestock turnout or if pastures are grazed permanently. Further monitoring of insect populations and vegetation dynamics should thus be carried out to study long‐term effects.

## AUTHOR CONTRIBUTIONS

Sebastian Seibold, Jörg Ewald, Martina Hofmann and Roland Baier conceptualized the study. Herbert Nickel, Johann Neumayer, Katharina Pospisil, Verena Styrnik, Denise Klein, Tobias Richter and Lisa Geres collected the data. Bernd Panassiti curated all raw data and prepared insect and plant traits. Valeria Trivellone created the phylogenetic trees for insects and plants. Bernd Panassiti conducted the analysis and visualized the results. Bernd Panassiti, Sebastian Seibold, Sebastian König and Valeria Trivellone wrote the first draft of the manuscript. All authors contributed critically to the drafts and gave final approval for publication.

## CONFLICT OF INTEREST STATEMENT

The authors declare no conflicts of interest.

## Supporting information


Appendix S1.

## Data Availability

Raw data to run the analyses are provided in Figshare as csv tables and an RData file in Panassiti (2025a), (2025b) at https://doi.org/10.6084/m9.figshare.27855306.v1 and https://doi.org/10.6084/m9.figshare.27855282.v1, respectively. Code (Panassiti, 2025c) is available in Zenodo at https://doi.org/10.5281/zenodo.17285119.
