# Supplementary material for: Effects of the timing of grazing on insect diversity and insect–plant interactions in mountain grasslands
Source: Ecol Appl. 2025 Nov 11;35(7):e70129. doi: 10.1002/eap.70129 (PMC12611450; doi:10.1002/eap.70129)
Supplement: Supplementary file 1 — Appendix S1. [file EAP-35-e70129-s001.pdf]

Journal: Ecological Applications

Effects of the timing of grazing on the diversity of insect communities and insect–plant interactions in mountain grasslands

Bernd Panassiti, Jörg Ewald, Martina Hofmann, Valeria Trivellone, Verena Styrnik, Herbert Nickel, Johann Neumayer, Katharina Pospisil, Denise Klein, Cynthia Tobisch, Sebastian König, Tobias Richter, Lisa Geres, Roland Baier, Sebastian Seibold

- Appendix S1 -

Study sites

The mountain pastures belong to the State of Bavaria and local farmers are allowed to use it for pasturing, often with a century-long tradition. The bedrock of the study sites is characterized by limestone, dolomite, or radiolarian rock with a mineral soil pH_{KCl} ranging from 3.42 - 6.96 (Tab. S1). The pasture vegetation is dominated by *Nardus stricta* (Poaceae) on acidic soils or Ericaceae (e.g. *Erica carnea*) and *Sesleria caerulea* (Poaceae) on neutral to alkaline soils (Ewald et al. 2024).

Table S1. Abiotic and biotic site conditions of the studied mountain pastures.

**site id**	**elevation**	**exposition**	**vegetation - plant communities - index species**	**geology**	**mineral soil pH_{KCl}**	**treatment size (ha)**	**Standard livestock unit[§] grazing days per hectare and per seasons (SLU × grazing days × ha^{-1} × grazing $season^{-1}$) [#]**
A001	780 - 840 m	SE	nutrient-poor grassland	dolomite, moraine	6.53	control: 0.09<br>early: 3<br>late: 3	90
A002	1400 - 1500 m	W (SW)	*Nardus stricta*	Dachstein limestone,<br>talus material	3.88	control: 0.09<br>early: 8<br>late: 19	51
A003	1100 - 1340 m	SE	Seslerio-Caricetum sempervirentis,<br>*Festuca rubra* agg.	Ramsau dolomite	6.96	control: 0.09<br>early: 13<br>late: 14	55
A004	1320 - 1380 m	N	*Festuca rubra* agg.	talus material,<br>Dachstein limestone	4.99	control: 0.09<br>early: 5<br>late: 5	45
A005	600 - 700 m	N	*Festuca rubra* agg.,<br>*Carex ferruginea*	talus material	5.83	control: 0.09<br>early: 11<br>late: 7	197
A006	1300 - 1540 m	SW	*Carex ferruginea*,<br>*Nardus stricta*,<br>*Festuca rubra* agg.	radiolarian rock,<br>red Lias limestone,<br>talus material	4.09	control: 0.09<br>early: 7<br>late: 6	120
A007	1620 - 1740 m	S	*Nardus stricta*	radiolarian rock	3.42	control: 0.09<br>early: 9<br>late: 10	177
A008	1440 - 1580 m	SW	*Nardus stricta*,<br>*Festuca rubra* agg.	moraine,<br>Dachstein limestone,<br>red Lias limestone	3.52	control: 0.09<br>early: 10<br>late: 6	140

[§] A standard livestock unit is defined as a cattle weight of 500 kg. [#] Means of early and late turnout treatments.

Study design

On each of the eight mountain pastures, we established three grazing treatments before the start of the grazing season in 2021, resulting in a total of 24 plots. Using electric fences, each pasture was divided into two halves with similar topography and vegetation and assigned one half to early and the other to late-turnout treatment. Furthermore, an ungrazed fenced area of approximately 900 m^2 was set up at each pasture as control. The livestock turnout dates in the early-turnout treatment were selected based on the vegetation development in each year (2021 and 2022), with grazing starting shortly after the snow melt as soon as the vegetation sustained livestock. Depending on the elevation of the pasture, early-turnout dates varied between early May and mid June (Tab. S2). Late-turnout dates in our study corresponded to the turnout dates of the past decades, which are legally fixed for each pasture in the regulations on the right of use. Late-turnout dates varied with elevation between the end of May and the end of July. These dates have not changed for decades and presumably reflect the phenology of vegetation before climate change. Late-turnout dates were on average three weeks later than early-turnout dates of the same pasture. The livestock was first moved to the early-turnout treatment and then moved to the late-turnout treatment. After the vegetation on the late-turnout plot was grazed, the livestock was either moved back to the early-turnout and then to the later-turnout treatment again, or the livestock was allowed to roam freely on the entire pasture (see Tab. S2 for turnout and turnover dates). All pastures were grazed exclusively by cattle, including dairy cows and heifers, with a carrying capacity of on average of 111 standard livestock unit grazing days per hectare and grazing season (Tab. S1).

Vegetation surveys

Vegetation surveys were carried out twice a year in 2021 and 2022. The first vegetation survey was carried out between mid April and mid June, and the second one between mid-

July and beginning of August. On each site, we randomly selected three vegetation plots in each of the turnout treatments (early and late grazing), and one vegetation plot in the control. The size of the quadratic vegetation plots was 9 m^2. Within these plots, species richness and cover of all vascular plants was recorded. To estimate plant cover, we used a modified Londo scale ranging from 0 to 100% (Londo 1976).

Livestock turnout dates

Table S2. Livestock turnout and turnover dates and insect sampling dates for the studied mountain pastures in the Berchtesgaden National Park in 2021 and 2022. Column names from column 3 are calendar weeks. Dates indicate start of grazing and insect sampling, respecitively. Grazing in early treatment plots is indicate by fields filled with orange color, in late turnout plots with blue color. The gray color indicates that grazing occurred over the entire area while white color indicates that no grazing occurred in the treatments. White fields with dates indicate dates when livestock was removed from the experimental plots. Some mountain pastures are not grazed for a period during the summer because the animals are grazing at higher elevations. The ungrazed control area was never grazed. Please note: “Ungrazed control” refers to permanently fenced plots with no grazing, whereas “no grazing” (white fields) indicates time periods when the grazed experimental plots (early and late) were temporarily ungrazed. * no pollinator sampling during summer onset, ** no pollinator sampling during summer peak.

year	site id	type	18	19	20	21	22	23	24	25	26	27	28	29	30	31	32	33	34	35	36	37	38	39	40	41
2021	A001	grazing	07.05.		17./22.05.		02.06.				01.07.		15.07. -	21.07.	28.07.			16.08.	26.08.						04.10.	12.10.
		pollinator										06.07.				03.08.										
		leafhopper										09.07.									06.09.					
	A002	grazing							19.06.					24.07.						30.08.		18.09.	25.09.			
		pollinator														07.08.				03.09.						
		leafhopper										09.07.								05.09.						
	A003	grazing			21.05.				18.06.				16.07.											02.10.		
		pollinator									28.06.					03.08.										
		leafhopper										07.07.								05.09.						
	A004	grazing								26.06.				19.07.				19.08.		03.09.						
		pollinator												22.07.						02.09.						
		leafhopper										07.07.								04.09.						
	A005	grazing			21.05.				20.06.			11.07.			01.08.					30.08.					09.10.	
		pollinator								23.06.				21./22.07.												
		leafhopper										08.07.								03.09.						
	A006	grazing							14.06.		28.06.	06.07.								30.08.			20.09.			
		pollinator										05.07.		24.07.												
		leafhopper										05./06.07.								04.09.						
	A007	grazing							19.06.		03.07.	11.07.							29.08.		11.09.					
		pollinator										10.07.		24.07.												
		leafhopper										06.07.								03.09.						
	A008	grazing						12.06.		24.06.	02.07.											18.09.				
		pollinator									29.06.	10.07.														
		leafhopper										06.07.								03.09.						

early livestock turnout

late livestock turnout

grazing over the entire area

no grazing

Table S2. Continued.

year	site_id		18	19	20	21	22	23	24	25	26	27	28	29	30	31	32	33	34	35	36	37	38	39	40	41
2022	A001	grazing	- excluded from experiment -																							
	A002	grazing							15.06.			09.07.						15.8.				16.09.				
		pollinator												21.07.					28.08.							
	A003	grazing			21.05.				17.06.				15.07.											01.10.		
		pollinator									27.06.			22.07.												
	A004	grazing								25.06.			15.07.				09.08.				09.09.					
		pollinator*															13.08.									
	A005	grazing		14.05.					13.06.			10.07.					12.08.				08.09.	17.09.		02.10.		
		pollinator							14./16.06.				14.07.													
	A006	grazing					03.06.		15.06.		01.07.								24.08.					01.10.		
		pollinator							15.06.			08.07.														
	A007	grazing				29.05.			19.06.			07.07.								03.09.	11.09.					
		pollinator**								26.06.																
	A008	grazing					04.06.			23.06.	02.07.											14.09.				
		pollinator									27.06.	09.07.														

- early livestock turnout
- late livestock turnout
- grazing over the entire area
- no grazing

Table S3. Traits used for insects and plants.

group	trait groups	traits	unit	description	reference
wild bee	Food plant specialization	Proboscis length	mm	numeric, min=3, max=16.2 , mean=6.9, sd=3.43, NA=3	J. Neumeyer, unpublished data
wild bee	Food plant specialization	Life strategy	-	categorical, habitat usage, generalist (16), specialist (11)	Westrich (2019)
wild bee	Reproduction and development	Body size	cm	numeric, min=5.5, max=18.7, mean=11.9, sd=4.06	Wiesbauer (2020)
wild bee	Reproduction and development	Voltinism	-	categorical, number of generations per year: 1 (23), 2 (3), NA (1)	Westrich (2019)
wild bee	Reproduction and development	Flight period	-	numeric, min=3, max=8, mean=5.8, sd=1.52	Westrich (2019)
wild bee	Reproduction and development	Social behaviour	-	categorical: parasitic (pa, 1), solitary (sl, 8), social (sc, 18)	Westrich (2019)
wild bee	Reproduction and development	Nest type	-	categorical, type of nests: in insect tunnels, e.g. dead wood (IP, 1), self produced (sH, 8), uses both type of nests (sH_IP, 1), existing below or above ground cavities/ structures, e.g. mouseholes, under stones, etc. (vH, 13)	Westrich (2019)
wild bee	Reproduction and development	Nest location	-	categorical, belowground (en, 13), aboveground (hy, 4), below and aboveground (en_hy, 8)	Westrich (2019)

Table S3. Continued.

butterfly	Food plant specialization	Feeding niche	-	categorical, monophagous (one genus, 18), oligophagous (one family, 25), polyphagous (>1 family, 18)	Settele et al. (1999)
butterfly	Dispersal and distribution	Wing length	cm	numeric, min=11, max=60, mean=23.9, sd=9.28	Higgins & Riley (1978)
butterfly	Dispersal and distribution	Migration propensity		categorical, highly sedentary (1) - highly migratory (8), 1 (1), 2 (2), 3 (14), 4 (19), 5 (4), 6 (5), 7 (1), 8 (2)	Settele et al. (1999)
butterfly	Dispersal and distribution	Distribution index	%	numeric, min=1.45, max=45.45, mean=26.3, sd=11.6, butterfly distribution in relation to total area of Europe	Kudrna (2002)
butterfly	Dispersal and distribution	Population density	ind/ha	numeric, average number of individuals per hectar, min= 0.06, max=160, mean= 29.4, sd= 36.5	Settele et al. (1999)
butterfly	Reproduction and development	Number of eggs	-	numeric, number eggs in a female at the beginning of egg laying period, min=23, max=445.5, mean=89.4, sd=75.9	Settele et al. (1999)
butterfly	Reproduction and development	Voltinism	-	categorical, number of generations per year, 1: 1 generation every 2 years (5), 2: 1 (30), 3: 1 or 2 (11), 4: 2 (5), 5: 2-3 (6) , 6: 3-4 (3)	Settele et al. (1999)
butterfly	Reproduction and development	Egg maturation	days	numeric, time between emergence and first oviposition, min=1.5, max=15.5, mean=6.10, sd=3.10	Settele et al. (1999)

Table S3. Continued.

butterfly	Reproduction and development	Hibernation stage	-	1=egg, 2-4=larval stages one to three, 5=pupa, 6=adult, 7=overwintering in warmer regions	Settele et al. (1999)
butterfly	Reproduction and development	Flight period	-	numerical, corresponds to the life span of adult butterflies, months are summed for multivoltine species, for overwintering adults only the flight period during the warm months (April – September) is considered, min=2, max=9, mean=4.73, sd=1.95	Settele et al. 2009
butterfly	Food plant specialization	Adult feeding	-	categorical, 16 categories with one or combinations of the following food sources used by adult butterflies: herb flower, ergot, shrub/tree flower, honeydew, sap, decaying plant, animal and mineral	European and North African (Maghreb) butterflies trait database; Middleton-Welling et al. (2020)
butterfly	Food plant specialization	Proboscis length	mm	numeric, min=5, max=27.1, mean=12.4, sd=4.18	Vajna et al. (2021)
leafhopper	Food plant specialization	Life strategy	-	categorical, generalist (19), specialist (80)	Nickel (2003)
leafhopper	Reproduction and development	Body length	cm	numeric, min=1.75, max=7.5, mean=3.78, sd=1.09	Biedermann & Niedringhaus (2009)
leafhopper	Dispersal and distribution	Pasture indicator	-	categorical, 0 (77), 1 (22)	Nickel (2003)
leafhopper	Food plant specialization	Diet width	-	categorical, m1 (20), m2 (17), o1 (36), o2 (7), po (19)	Nickel (2003)

Table S3. Continued.

leafhopper	Dispersal and distribution	Aridity range	-	categorical, 1 (20), 2 (28), 3 (15)	Nickel (2003)
leafhopper	Reproduction and development	Overwintering	-	categorical, egg (70), nymph (17), adult (12)	Biedermann & Niedringhaus (2009)
leafhopper	Reproduction and development	Voltinism	-	categorical, 1 (56), 1-2 (13), 2 (30)	Biedermann & Niedringhaus (2009)
plant - pollinator network	Pollinator attraction	Flower size	mm	numerical, min=9.41, max=307, mean=37.4, sd=58.5, NA=36	FReD database; Arnold et al. (2010)
plant - pollinator network	Pollinator attraction	Display size	mm	numerical, min=0.91, max=20.3, mean=7.15, sd=6.73, NA=36	FReD database; Arnold et al. (2010)
plant - pollinator network	Pollinator attraction	UV reflectance periphery	-	categorical, 1 (18), 2 (8), 3 (6), 4(4), 5 (6)	FReD database; Arnold et al. (2010)
plant - pollinator network	Pollinator attraction	UV reflectance patterns	-	categorical, 0 (24), 1 (17)	FReD database; Arnold et al. (2010)
plant - pollinator network	Pollinator attraction	Nectar tube depth	mm	numerical, min=0, max=16.5, mean=5.37, sd=5.06, NA=36	FReD database; Arnold et al. (2010)
plant - pollinator network	Pollinator attraction	Flower color	-	categorical, blue (3), braun (1), green (2), orange (1), pink (9), red (1), violet (11), white (12), yellow (16)	FReD database; Arnold et al. (2010)
plant - phytophagous network	Morphology	Stem specific density (SSD, stem dry mass per stem fresh volume) or wood density	mg/mm^3	numerical, min=0.18, max=0.29, mean=0.23, sd=0.05, NA=5	TRY database; Kattge et al. (2020)
plant - phytophagous network	Quality	Plant nitrogen(N) fixation capacity	-	categorical, no (6), yes (6)	TRY database; Kattge et al. (2020)

Table S3. Continued.

plant - phytophagous network	Quality	Leaf nitrogen (N) content per leaf dry mass (%)	%	numerical, min=1.3, max=4.22, mean=1.96, sd=0.75	TRY database; Kattge et al. (2020)
plant - phytophagous network	Quality	Leaf phosphorus (P) content per leaf dry mass (%)	%	numerical, min=0.0004, max=0.43, mean=0.15, sd=0.107	TRY database; Kattge et al. (2020)
plant - phytophagous network	Quality	Leaf nitrogen (N) content per leaf area	mmol/ m^2	numerical, min=1.03, max=81.8, mean=12.9, sd=30.3, NA=5	TRY database; Kattge et al. (2020)
plant - phytophagous network	Biomass	Leaf dry mass (single leaf) (mg)	mg	numerical, min=19.2, max=95.9, mean=42.8, sd=23.7	TRY database (Kattge et al. 2020)
plant - phytophagous network	Quality	Leaf nitrogen/phosphorus (N/P) ratio	g/g	numerical, min=9.18, max=16.9, mean=12.2, sd=2.99, NA=3	TRY database (Kattge et al. 2020)
plant - phytophagous network	Morphology	Leaf length	cm	numerical, min=6.5, max=30.6, mean=18.32, sd=9.31, NA=6	TRY database (Kattge et al. 2020)
plant - phytophagous network	Quality	Leaf carbon/nitrogen (C/N) ratio	-	numerical, min=10.7, max=30.7, mean=24.5, sd=5.35, NA=1	TRY database (Kattge et al. 2020)
plant - phytophagous network	Morphology	Plant height vegetative	cm	numerical, min=15.3, max=96.9, mean=37.5, sd=20.9	TRY database (Kattge et al. 2020)
plant - phytophagous network	Morphology	Leaf area (in case of compound leaves: leaf, petiole excluded)	cm^2	numerical, min=2.25, max=50.9, mean=27.5, sd=14.3, NA=3	TRY database (Kattge et al. 2020)
plant - phytophagous network	Morphology	Leaf area per leaf dry mass (specific leaf area, SLA or 1/LMA): petiole excluded	cm^2/g	numerical, min=126, max=401, mean=264, sd=86.4, NA=3	TRY database (Kattge et al. 2020)
plant - phytophagous network	Biomass	Leaf cell wall mass per leaf dry mass	%	numerical, min=46.9, max=67, mean=58.7, sd=8.57, NA=8	TRY database (Kattge et al. 2020)

Statistical analyses

The *brm* function from the BRMS R package uses the Stan platform (v. 2.32.3, Stan Development Team 2023) that implements the No-U-Turn Sampler, an extension to the Hamiltonian Monte Carlo algorithm that eliminates the need to set a number of leapfrog steps (Hoffman and Gelman 2014). We chose 5,000 iteration steps and four chains. The four chains were considered to have converged when the R-hat convergence diagnostic was <1.05 (Gelman 2014). Moreover, we controlled the sampler behavior to avoid divergent transitions after warm-up and hence biased posterior draws. Therefore, we slowed the sampling speed using the argument 'adapt_delta' set to 0.9999 and increased the tree depth to be evaluated by setting the argument 'max treedepth' = 15. Fixed effects priors were normally distributed and centered around zero. We set the fixed effect prior to be mildly informative (standard deviation = 10) which applies a shrinkage comparable to a ridge regression. For random effects priors default settings were used, i.e. half student-t priors with 3 degrees of freedom with a scale parameter of 2.5. Across all fitted GLMM and SEM models, the Bayesian R^2 ranged from 0.25 to 0.70 and 0.20 to 0.67. We used Pareto smoothed importance-sampling to check the reliability of the estimates (Vehtari et al. 2017). The Bayesian version of R^2 conditioning on fixed as well as random effects was calculated using the function 'bayes_R' from the *brms* package (Gelman et al. 2019). We also estimated elpd_loo for each model, the Bayesian Leave-One-Out Cross-Validation estimate of the expected log pointwise predictive density (Vehtari et al. 2017). Values can be negative or positive and can be used to evalute predictive model performance. Higher ELPD-LOO values are better, as they indicate that the model assigns higher probability to unseen data (i.e., better predictive performance).

Results

Vegetation surveys

In the vegetation plots, in total 323 vascular plant species were identified with an average of 46 plant species per plot (±12.1). Plant species richness, as well as herb and grass cover, showed no significant differences between control and grazed plots, nor between early and late turnout treatments (Fig. S1).

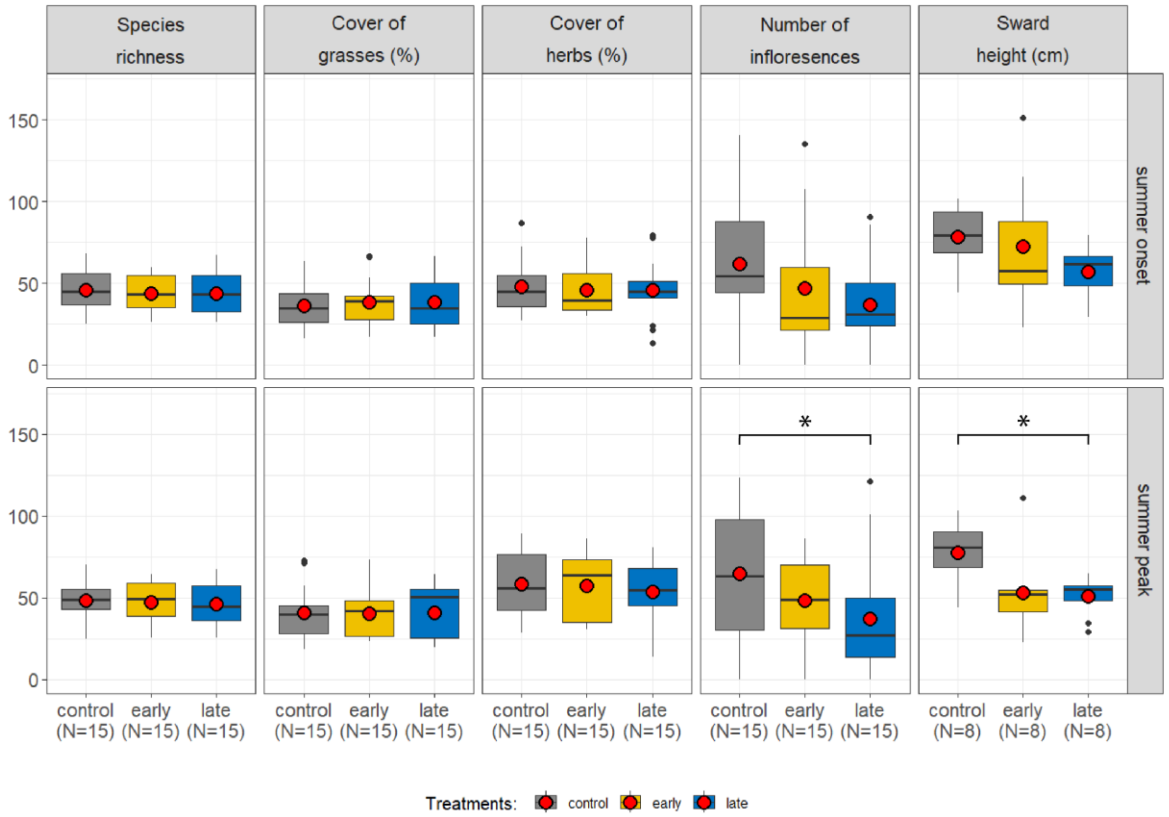

Figure S1. Overview of plant parameters recorded in the field (species richness, cover of herbs and grasses, number of inflorescences, sward height) for 2 survey periods (summer onset and summer peak). We surveyed eight mountain pastures in two years (2021, 2022). Due to logistic reasons, 1 alpine pasture was excluded in 2022. The number of inflorescences were divided by 10. Stars indicate significant differences between treatments based on Tukey's multiple comparisons of means ($p < 0.05$).

Q1) Effect of livestock turnout dates on wild bees, butterflies, leafhoppers

Pollinators were not significantly influenced by plant SR and plant cover (Fig. S2). However, for leafhoppers we found that herb cover increased functional diversity during summer onset, and taxonomic, phylogenetic and functional diversity during summer peak (Fig. S2).

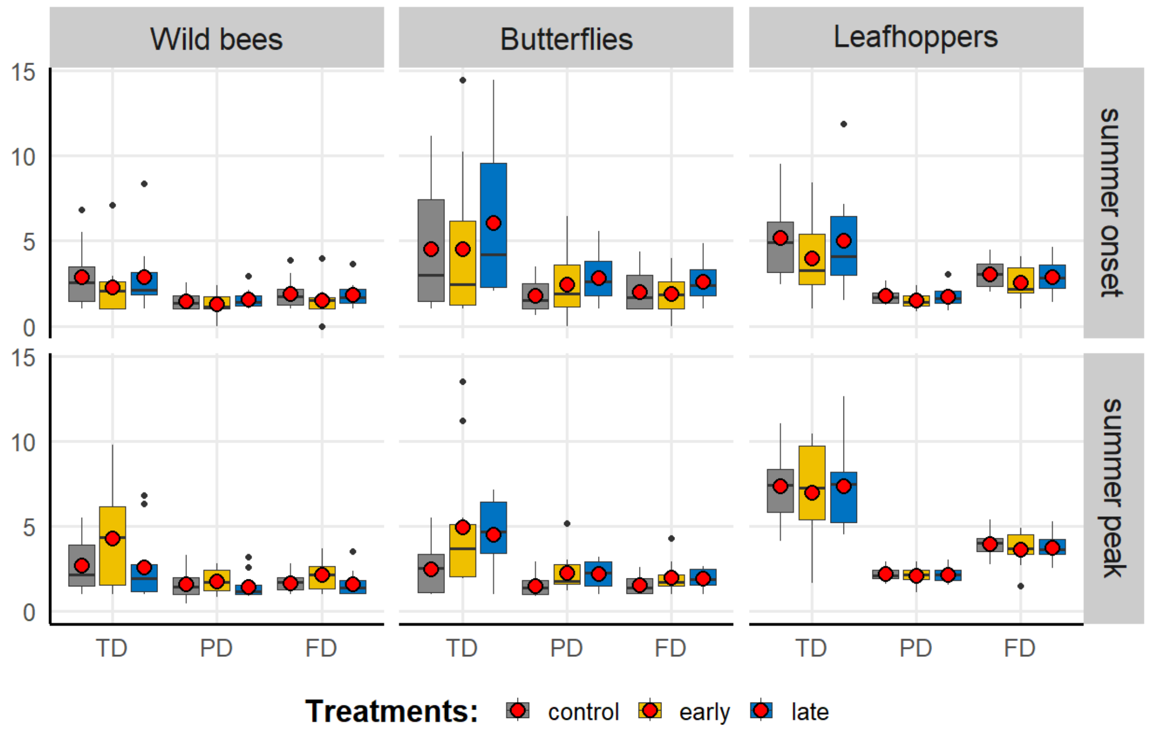

Figure S2. Estimated taxonomic, phylogenetic and functional alpha diversity (TD, PD and FD) for each treatment and sampling period for wild bees, butterflies and leafhoppers. Diversity measures are based on Hill number q = 0 and were standardized at a sample coverage of 0.7. Red dot indicates mean values. Number of observations in treatments control, early and late are 12, 9, 10 for wild bees during summer onset and 14, 11, 12 during summer peak; and 14, 10, 11 for butterflies during summer onset and 12, 10, 11 during summer peak. For leafhoppers the number of observations was always eight. Please note, wild bees and butterflies were sampled in years 2021 and 2022, and leafhoppers only in 2021.

GLMM with management pedictors

Tables S4 and S5 show GLMM model results with management as predictor. Management was defined as early and late livestock turnout.

Table S4. GLMM model coefficients. Models were fitted for 3 insect groups (wild bees, butterflies, leafhoppers) and for 3 diversity types: taxonomic (TD), phylogenetic (PD), and functional (FD) diversity. Numbers in brackets indicates standard deviations.

GLMM model type	diversity type	Intercept	summer peak	year 2022	elevation	summer onset : Early	summer peak : Early	summer onset : Late	summer peak : Late
Wild bee	TD	2.01 (0.684)	0.206 (0.775)	1.07 (0.456)	-0.629 (0.454)	-0.96 (0.767)	0.681 (0.774)	-0.32 (0.772)	-0.497 (0.768)
Butterfly	TD	4.21 (0.998)	-2.43 (1.29)	0.6 (0.76)	-1.64 (0.462)	-0.737 (1.33)	1.44 (1.29)	0.547 (1.33)	1.34 (1.27)
Leafhopper	TD	5.1 (1.09)	2.21 (0.913)		-0.773 (0.946)	-1.18 (0.908)	-0.388 (0.904)	-0.155 (0.914)	-0.00178 (0.91)
Wild bee	PD	1.17 (0.258)	0.278 (0.304)	0.267 (0.182)	-0.248 (0.147)	-0.426 (0.304)	-0.159 (0.303)	-0.0927 (0.313)	-0.33 (0.307)
Butterfly	PD	1.64 (0.395)	-0.468 (0.51)	0.236 (0.304)	-0.587 (0.186)	0.261 (0.522)	0.356 (0.509)	0.617 (0.52)	0.428 (0.508)
Leafhopper	PD	1.76 (0.238)	0.449 (0.182)		-0.154 (0.202)	-0.251 (0.188)	-0.1 (0.185)	-0.0163 (0.185)	-0.039 (0.184)
Wild bee	FD	1.4 (0.302)	0.0824 (0.332)	0.385 (0.201)	-0.354 (0.191)	-0.561 (0.333)	-0.021 (0.33)	-0.194 (0.336)	-0.295 (0.323)
Butterfly	FD	1.9 (0.317)	-0.611 (0.405)	0.07 (0.239)	-0.581 (0.155)	-0.329 (0.409)	0.0967 (0.398)	0.266 (0.409)	0.166 (0.404)
Leafhopper	FD	3.07 (0.442)	0.924 (0.353)		-0.155 (0.358)	-0.521 (0.353)	-0.345 (0.347)	-0.194 (0.354)	-0.224 (0.348)

Table S5. GLMM model performances. Models were fitted for 3 insect groups (wild bees, butterflies, leafhoppers) and for 3 diversity types: taxonomic (TD), phylogenetic (PD), and functional (FD) diversity. We computed the Bayesian version of R^2. elpd_loo is the Bayesian LOO estimate of the expected log pointwise predictive density. Higher elpd_loo values indicate better predictive performance.

GLMM model type	diversity type	bayes R^2				elpd_loo
		Estimate	Est.Error	Q2.5	Q97.5	
Wild bee	TD	0.30	0.0702	0.16	0.43	-183
Butterfly	TD	0.29	0.0635	0.16	0.40	-222
Leafhopper	TD	0.66	0.0592	0.52	0.75	-104
Wild bee	PD	0.25	0.0669	0.12	0.38	-106
Butterfly	PD	0.26	0.0636	0.14	0.38	-145
Leafhopper	PD	0.65	0.0599	0.51	0.74	-27.0
Wild bee	FD	0.30	0.0687	0.16	0.43	-113
Butterfly	FD	0.32	0.0650	0.19	0.44	-126
Leafhopper	FD	0.63	0.0641	0.48	0.73	-57.5

GLMM with vegetation pedictors

Tables S6 and S7 show GLMM model results with vegetation as predictor. For wild bee and butterfly models we used the number of inflorescences as predictor, and for leafhoppers the sward height.

Table S6. GLMM model coefficients. Models were fitted for 3 insect groups (wild bees, butterflies, leafhoppers) and for 3 diversity types: taxonomic (TD), phylogenetic (PD), and functional (FD) diversity. Plant is the number of inflorescences for wild bees and butterflies, and sward height for leafhoppers. Numbers in brackets indicates standard deviations.

GLMM model type	diversity type	Intercept	summer peak	year 2022	elevation	summer onset : plant*	summer peak : plant*
Wild bee	TD	0.72 (0.199)	-0.171 (0.226)	0.235 (0.123)	-0.211 (0.0996)	0.000191 (0.000277)	0.000855 (0.000233)
Butterfly	TD	1.53 (0.26)	-0.462 (0.298)	0.0684 (0.162)	-0.426 (0.123)	-0.000343 (0.000369)	2.9e-05 (0.000299)
Leafhopper	TD	1.95 (0.241)	-0.0602 (0.244)		-0.16 (0.17)	-0.00455 (0.00237)	0.00267 (0.00309)
Wild bee	PD	0.613 (0.141)	-0.1 (0.16)	0.106 (0.0858)	-0.128 (0.072)	1.38e-05 (0.000197)	0.000438 (0.000159)
Butterfly	PD	1.04 (0.185)	-0.292 (0.21)	0.081 (0.115)	-0.25 (0.0841)	-0.000205 (0.000258)	5.4e-05 (0.000208)
Leafhopper	PD	1.12 (0.102)	-0.0478 (0.0977)		-0.0702 (0.0713)	-0.00223 (0.000979)	0.00114 (0.00126)
Wild bee	FD	0.587 (0.146)	-0.0687 (0.162)	0.135 (0.088)	-0.156 (0.0724)	0.000161 (2e-04)	0.000505 (0.000165)
Butterfly	FD	1 (0.164)	-0.325 (0.188)	0.0249 (0.1)	-0.267 (0.0801)	-0.000117 (0.000233)	0.000157 (0.000185)
Leafhopper	FD	1.51 (0.141)	-0.0817 (0.148)		-0.0578 (0.0936)	-0.00301 (0.0015)	0.00187 (0.00191)

*plant = number of inflorescences (wild bee, butterfly) or sward height (leafhopper)

Table S7. GLMM model performances. Models were fitted for 3 insect groups (wild bees, butterflies, leafhoppers) and for 3 diversity types: taxonomic (TD), phylogenetic (PD), and functional (FD) diversity. We computed the Bayesian version of R^2. elpd_loo is the Bayesian LOO estimate of the expected log pointwise predictive density. Higher elpd_loo values indicate better predictive performance.

GLMM model type	diversity type	bayes R^2				elpd_loo
		Estimate	Est.Error	Q2.5	Q97.5	
Wild bee	TD	0.38	0.0658	0.24	0.50	-71.6
Butterfly	TD	0.32	0.0672	0.18	0.44	-91.5
Leafhopper	TD	0.67	0.0571	0.53	0.75	-14.5
Wild bee	PD	0.29	0.0688	0.16	0.42	-42.2
Butterfly	PD	0.26	0.0675	0.13	0.39	-62.8
Leafhopper	PD	0.69	0.0516	0.57	0.76	28.7
Wild bee	FD	0.34	0.0678	0.20	0.46	-44.2
Butterfly	FD	0.33	0.0677	0.19	0.45	-53.0
Leafhopper	FD	0.62	0.0661	0.46	0.71	7.79

Q2) Direct and indirect effects of livestock turnout dates on wild bees, butterflies, leafhoppers

Table S8. SEM standardized path-coefficients for SEM based on taxonomic diversity (TD). SEMs were fitted for 3 insect groups (wild bees, butterflies, leafhoppers). Insect model responses were based on taxonomic species-richness based diversity (TD). Plant model responses are the number of inflorescences for wild bees and butterflies, and sward height for leafhoppers.

SEM model type	Model	variable	mean	sd	q5	q95
Wild bee	Insect	Intercept	0.436	0.367	-0.162	1.044
Wild bee	Insect	Summer peak	-0.253	0.367	-0.856	0.347
Wild bee	Insect	Number of inflorescences	0.001	0.000	0.001	0.002
Wild bee	Insect	Year 2022	0.327	0.228	-0.050	0.699
Wild bee	Insect	Elevation	-0.070	0.154	-0.319	0.173
Wild bee	Insect	Summer onset : Early turnout	-0.179	0.404	-0.843	0.491
Wild bee	Insect	Summer peak : Early turnout	0.947	0.377	0.318	1.566
Wild bee	Insect	Summer onset : Late turnout	0.138	0.392	-0.498	0.798
Wild bee	Insect	Summer peak : Late turnout	0.302	0.386	-0.327	0.927
Wild bee	Plant	Intercept	1.544	0.332	1.000	2.087
Wild bee	Plant	Summer peak	0.495	0.307	-0.013	0.995
Wild bee	Plant	Year 2022	0.115	0.199	-0.211	0.445
Wild bee	Plant	Elevation	-0.117	0.262	-0.536	0.308
Wild bee	Plant	Summer onset : Early turnout	-0.234	0.351	-0.814	0.328
Wild bee	Plant	Summer peak : Early turnout	-0.567	0.323	-1.100	-0.038
Wild bee	Plant	Summer onset : Late turnout	-0.422	0.338	-0.973	0.151
Wild bee	Plant	Summer peak : Late turnout	-0.946	0.309	-1.445	-0.433
Butterfly	Insect	Intercept	1.442	0.377	0.809	2.056
Butterfly	Insect	Summer peak	-0.573	0.392	-1.229	0.059
Butterfly	Insect	Number of inflorescences	0.000	0.000	-0.001	0.000
Butterfly	Insect	Year 2022	0.089	0.237	-0.296	0.481
Butterfly	Insect	Elevation	-0.344	0.148	-0.582	-0.109
Butterfly	Insect	Summer onset : Early turnout	-0.041	0.401	-0.715	0.614
Butterfly	Insect	Summer peak : Early turnout	0.530	0.428	-0.167	1.242
Butterfly	Insect	Summer onset : Late turnout	0.258	0.397	-0.395	0.919
Butterfly	Insect	Summer peak : Late turnout	0.376	0.432	-0.330	1.087
Butterfly	Plant	Intercept	1.816	0.347	1.261	2.378
Butterfly	Plant	Summer peak	0.406	0.313	-0.109	0.928
Butterfly	Plant	Year 2022	-0.119	0.201	-0.447	0.217
Butterfly	Plant	Elevation	-0.091	0.304	-0.569	0.396
Butterfly	Plant	Summer onset : Early turnout	-0.310	0.332	-0.854	0.232
Butterfly	Plant	Summer peak : Early turnout	-0.677	0.335	-1.229	-0.131
Butterfly	Plant	Summer onset : Late turnout	-0.582	0.316	-1.095	-0.056
Butterfly	Plant	Summer peak : Late turnout	-1.337	0.326	-1.867	-0.811
Leafhopper	Insect	Intercept	1.994	0.559	1.085	2.904
Leafhopper	Insect	Summer peak	0.738	0.313	0.224	1.254
Leafhopper	Insect	Sward height	-0.003	0.005	-0.012	0.005
Leafhopper	Insect	Elevation	-0.273	0.330	-0.806	0.250
Leafhopper	Insect	Summer onset : Early turnout	-0.423	0.317	-0.947	0.101
Leafhopper	Insect	Summer peak : Early turnout	-0.204	0.334	-0.741	0.345
Leafhopper	Insect	Summer onset : Late turnout	-0.124	0.336	-0.670	0.423
Leafhopper	Insect	Summer peak : Late turnout	-0.084	0.340	-0.648	0.472
Leafhopper	Plant	Intercept	3.009	0.365	2.413	3.605
Leafhopper	Plant	Summer peak	-0.025	0.385	-0.658	0.609
Leafhopper	Plant	Elevation	-0.039	0.291	-0.525	0.406
Leafhopper	Plant	Summer onset : Early turnout	-0.228	0.387	-0.852	0.398
Leafhopper	Plant	Summer peak : Early turnout	-0.958	0.386	-1.571	-0.328
Leafhopper	Plant	Summer onset : Late turnout	-0.823	0.383	-1.453	-0.210
Leafhopper	Plant	Summer peak : Late turnout	-1.043	0.387	-1.692	-0.406

Table S9. SEM standardized path-coefficients for SEM based on phylogenetic diversity (PD). SEMs were fitted for 3 insect groups (wild bees, butterflies, leafhoppers). Insect model responses were based on phylogenetic diversity (TD). Plant model responses are the number of inflorescences for wild bees and butterflies, and sward height for leafhoppers.

SEM model type	Model	variable	mean	sd	q5	q95
Wild bee	Insect	Intercept	1.724	0.415	1.041	2.406
Wild bee	Insect	Summer peak	0.045	0.422	-0.649	0.722
Wild bee	Insect	Number of inflorescences	0.001	0.000	0.000	0.001
Wild bee	Insect	Year 2022	0.071	0.251	-0.337	0.496
Wild bee	Insect	Elevation	-0.013	0.164	-0.271	0.244
Wild bee	Insect	Summer onset : Early turnout	-0.221	0.466	-0.977	0.567
Wild bee	Insect	Summer peak : Early turnout	0.453	0.430	-0.258	1.169
Wild bee	Insect	Summer onset : Late turnout	0.215	0.455	-0.533	0.960
Wild bee	Insect	Summer peak : Late turnout	0.059	0.440	-0.662	0.778
Wild bee	Plant	Intercept	1.549	0.328	1.009	2.086
Wild bee	Plant	Summer peak	0.489	0.304	-0.019	0.981
Wild bee	Plant	Year 2022	0.109	0.198	-0.214	0.440
Wild bee	Plant	Elevation	-0.123	0.262	-0.547	0.301
Wild bee	Plant	Summer onset : Early turnout	-0.236	0.349	-0.810	0.335
Wild bee	Plant	Summer peak : Early turnout	-0.560	0.310	-1.071	-0.052
Wild bee	Plant	Summer onset : Late turnout	-0.418	0.342	-0.980	0.153
Wild bee	Plant	Summer peak : Late turnout	-0.938	0.305	-1.444	-0.445
Butterfly	Insect	Intercept	1.671	0.398	1.022	2.337
Butterfly	Insect	Summer peak	-0.221	0.398	-0.876	0.428
Butterfly	Insect	Number of inflorescences	0.000	0.000	-0.001	0.000
Butterfly	Insect	Year 2022	0.043	0.244	-0.363	0.450
Butterfly	Insect	Elevation	-0.264	0.158	-0.517	-0.017
Butterfly	Insect	Summer onset : Early turnout	0.490	0.412	-0.181	1.174
Butterfly	Insect	Summer peak : Early turnout	0.471	0.439	-0.250	1.195
Butterfly	Insect	Summer onset : Late turnout	0.659	0.413	-0.020	1.335
Butterfly	Insect	Summer peak : Late turnout	0.373	0.457	-0.384	1.133
Butterfly	Plant	Intercept	1.828	0.351	1.266	2.403
Butterfly	Plant	Summer peak	0.405	0.319	-0.134	0.921
Butterfly	Plant	Year 2022	-0.125	0.202	-0.466	0.201
Butterfly	Plant	Elevation	-0.085	0.298	-0.556	0.402
Butterfly	Plant	Summer onset : Early turnout	-0.312	0.333	-0.860	0.235
Butterfly	Plant	Summer peak : Early turnout	-0.682	0.343	-1.246	-0.121
Butterfly	Plant	Summer onset : Late turnout	-0.585	0.319	-1.106	-0.068
Butterfly	Plant	Summer peak : Late turnout	-1.344	0.335	-1.887	-0.802
Leafhopper	Insect	Intercept	3.568	0.606	2.595	4.575
Leafhopper	Insect	Summer peak	0.777	0.314	0.260	1.292
Leafhopper	Insect	Sward height	-0.007	0.005	-0.016	0.002
Leafhopper	Insect	Elevation	-0.302	0.376	-0.933	0.281
Leafhopper	Insect	Summer onset : Early turnout	-0.474	0.318	-0.999	0.034
Leafhopper	Insect	Summer peak : Early turnout	-0.343	0.346	-0.901	0.224
Leafhopper	Insect	Summer onset : Late turnout	-0.167	0.334	-0.703	0.374
Leafhopper	Insect	Summer peak : Late turnout	-0.250	0.349	-0.814	0.329
Leafhopper	Plant	Intercept	3.004	0.361	2.404	3.595
Leafhopper	Plant	Summer peak	-0.030	0.393	-0.665	0.624
Leafhopper	Plant	Elevation	-0.035	0.286	-0.517	0.407
Leafhopper	Plant	Summer onset : Early turnout	-0.228	0.381	-0.848	0.405
Leafhopper	Plant	Summer peak : Early turnout	-0.939	0.385	-1.577	-0.303
Leafhopper	Plant	Summer onset : Late turnout	-0.816	0.384	-1.447	-0.189
Leafhopper	Plant	Summer peak : Late turnout	-1.032	0.391	-1.669	-0.375

Table S10. SEM standardized path-coefficients for SEM based on functional diversity (PD). SEMs were fitted for 3 insect groups (wild bees, butterflies, leafhoppers). Insect model responses were based on functional diversity (TD). Plant model responses are the number of inflorescences for wild bees and butterflies, and sward height for leafhoppers.

SEM model type	Model	variable	mean	sd	q5	q95
Wild bee	Insect	Intercept	1.565	0.382	0.936	2.182
Wild bee	Insect	Summer peak	-0.393	0.383	-1.007	0.238
Wild bee	Insect	Number of inflorescences	0.001	0.000	0.001	0.002
Wild bee	Insect	Year 2022	0.155	0.240	-0.226	0.545
Wild bee	Insect	Elevation	-0.094	0.154	-0.340	0.146
Wild bee	Insect	Summer onset : Early turnout	-0.322	0.423	-1.014	0.373
Wild bee	Insect	Summer peak : Early turnout	0.747	0.399	0.089	1.392
Wild bee	Insect	Summer onset : Late turnout	0.120	0.415	-0.565	0.809
Wild bee	Insect	Summer peak : Late turnout	0.300	0.394	-0.343	0.955
Wild bee	Plant	Intercept	1.549	0.334	0.996	2.091
Wild bee	Plant	Summer peak	0.491	0.311	-0.023	1.002
Wild bee	Plant	Year 2022	0.110	0.200	-0.219	0.437
Wild bee	Plant	Elevation	-0.122	0.255	-0.545	0.288
Wild bee	Plant	Summer onset : Early turnout	-0.232	0.351	-0.796	0.339
Wild bee	Plant	Summer peak : Early turnout	-0.569	0.321	-1.099	-0.044
Wild bee	Plant	Summer onset : Late turnout	-0.417	0.346	-0.985	0.147
Wild bee	Plant	Summer peak : Late turnout	-0.946	0.311	-1.466	-0.440
Butterfly	Insect	Intercept	2.218	0.387	1.588	2.845
Butterfly	Insect	Summer peak	-0.478	0.393	-1.126	0.170
Butterfly	Insect	Number of inflorescences	0.000	0.000	-0.001	0.000
Butterfly	Insect	Year 2022	-0.100	0.238	-0.486	0.293
Butterfly	Insect	Elevation	-0.363	0.158	-0.617	-0.117
Butterfly	Insect	Summer onset : Early turnout	-0.063	0.406	-0.726	0.607
Butterfly	Insect	Summer peak : Early turnout	0.321	0.436	-0.389	1.042
Butterfly	Insect	Summer onset : Late turnout	0.515	0.398	-0.133	1.168
Butterfly	Insect	Summer peak : Late turnout	0.260	0.446	-0.476	0.998
Butterfly	Plant	Intercept	1.822	0.346	1.249	2.384
Butterfly	Plant	Summer peak	0.405	0.319	-0.128	0.926
Butterfly	Plant	Year 2022	-0.127	0.201	-0.450	0.206
Butterfly	Plant	Elevation	-0.087	0.299	-0.565	0.397
Butterfly	Plant	Summer onset : Early turnout	-0.311	0.332	-0.860	0.238
Butterfly	Plant	Summer peak : Early turnout	-0.678	0.337	-1.237	-0.140
Butterfly	Plant	Summer onset : Late turnout	-0.587	0.317	-1.120	-0.064
Butterfly	Plant	Summer peak : Late turnout	-1.335	0.329	-1.873	-0.793
Leafhopper	Insect	Intercept	3.449	0.605	2.455	4.435
Leafhopper	Insect	Summer peak	0.862	0.323	0.323	1.387
Leafhopper	Insect	Sward height	-0.007	0.006	-0.016	0.002
Leafhopper	Insect	Elevation	-0.170	0.351	-0.750	0.383
Leafhopper	Insect	Summer onset : Early turnout	-0.527	0.323	-1.059	0.005
Leafhopper	Insect	Summer peak : Early turnout	-0.496	0.344	-1.071	0.076
Leafhopper	Insect	Summer onset : Late turnout	-0.343	0.350	-0.913	0.231
Leafhopper	Insect	Summer peak : Late turnout	-0.408	0.359	-0.996	0.183
Leafhopper	Plant	Intercept	3.009	0.362	2.400	3.585
Leafhopper	Plant	Summer peak	-0.029	0.388	-0.672	0.608
Leafhopper	Plant	Elevation	-0.037	0.279	-0.493	0.402
Leafhopper	Plant	Summer onset : Early turnout	-0.225	0.383	-0.848	0.393
Leafhopper	Plant	Summer peak : Early turnout	-0.953	0.386	-1.583	-0.325
Leafhopper	Plant	Summer onset : Late turnout	-0.832	0.383	-1.467	-0.201
Leafhopper	Plant	Summer peak : Late turnout	-1.034	0.389	-1.662	-0.387

Performance of SEM models

Table S11. SEM model results. Models were fitted for 3 insect groups (wild bees, butterflies, leafhoppers) and for 3 diversity types: taxonomic (TD), phylogenetic (PD), and functional (FD) diversity. Each SEM was included a plant (number of inflorescences or sward height) and an insect part. We computed the Bayesian version of R^2. elpd_loo is the Bayesian LOO estimate of the expected log pointwise predictive density. Higher elpd_loo values indicate better predictive performance.

SEM model type	diversity type	response	bayes R^2				elpd_loo
			Estimate	Est.Error	Q2.5	Q97.5	
Wild bee	TD	Wild bee SR	0.34	0.0672	0.19	0.46	-635
Wild bee	TD	Number of inflorescences	0.39	0.0739	0.23	0.52	
Butterfly	TD	Butterfly SR	0.27	0.0656	0.14	0.39	-677
Butterfly	TD	Number of inflorescences	0.48	0.0666	0.33	0.59	
Leafhopper	TD	Leafhopper SR	0.67	0.0583	0.53	0.75	-328
Leafhopper	TD	Sward height	0.50	0.0834	0.30	0.63	
Wild bee	PD	Wild bee SR	0.20	0.0611	0.08	0.32	-565
Wild bee	PD	Number of inflorescences	0.39	0.0739	0.23	0.52	
Butterfly	PD	Butterfly SR	0.25	0.0665	0.12	0.38	-606
Butterfly	PD	Number of inflorescences	0.48	0.0668	0.33	0.60	
Leafhopper	PD	Leafhopper SR	0.67	0.0565	0.53	0.75	-250
Leafhopper	PD	Sward height	0.50	0.0840	0.30	0.63	
Wild bee	FD	Wild bee SR	0.29	0.0689	0.15	0.42	-568
Wild bee	FD	Number of inflorescences	0.39	0.0740	0.24	0.52	
Butterfly	FD	Butterfly SR	0.28	0.0664	0.15	0.4	-587
Butterfly	FD	Number of inflorescences	0.48	0.0668	0.33	0.59	
Leafhopper	FD	Leafhopper SR	0.65	0.0585	0.51	0.74	-284
Leafhopper	FD	Sward height	0.50	0.0817	0.31	0.63	

SEM for phylogenetic diversity (PD)

Figure S3. Structural equation models (SEMs) showing direct and indirect effects on the phylogenetic diversity (PD) of (A) wildbees, (B) butterflies and (C) leafhoppers. Insects were collected in two sampling times (summer onset and summer peak). Treatments included early and late livestock turnout (early vs. late). Indirect effects of elevation and treatment via the number of inflorescences were tested for wild bees and butterflies and via sward height for leafhoppers. Numbers next to arrows indicate standardized path coefficients (e = early turnout, l = late turnout). Results of hypothesis tests are indicated by different arrow types. We tested that (1) the coefficients of early turnout are greater than of late turnout, (2) the elevation coefficient unequals zero, (3) the coefficient of the number of inflorescences unequals zero, and (4) the sward height coefficient unequals zero. Posterior probabilities of hypothesis tests with P>=0.95 are indicated by a bold arrow, still notable effects (0.8 < P < 0.95) by an arrow with a continuous line, and less noteworthy effects (P <= 0.8) by a dashed arrow. Original wild bee and leafhopper images provided by Pexels were modified and used under the Pexels License. The original butterfly image was provided by Katharina Pospisil and subsequently modified.

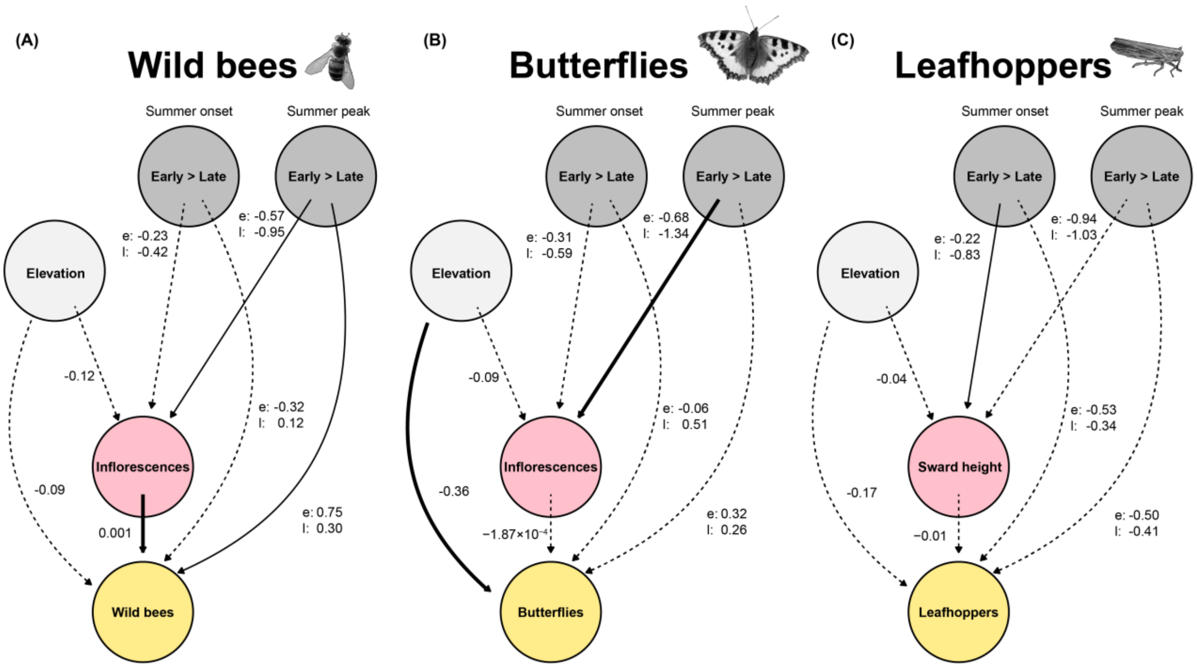

SEM for functional diversity (FD)

Figure S4. Structural equation models (SEMs) showing direct and indirect effects on the functional diversity (FD) of (A) wildbees, (B) butterflies and (C) leafhoppers. Insects were collected in two sampling times (summer onset and summer peak). Treatments included early and late livestock turnout (early vs. late). Indirect effects of elevation and treatment via the number of inflorescences were tested for wild bees and butterflies and via sward height for leafhoppers. Numbers next to arrows indicate standardized path coefficients (e = early turnout, l = late turnout). Results of hypothesis tests are indicated by different arrow types. We tested that (1) the coefficients of early turnout are greater than of late turnout, (2) the elevation coefficient unequals zero, (3) the coefficient of the number of inflorescences unequals zero, and (4) the sward height coefficient unequals zero. Posterior probabilities of hypothesis tests with P>=0.95 are indicated by a bold arrow, still notable effects (0.8 < P < 0.95) by an arrow with a continuous line, and less noteworthy effects (P <= 0.8) by a dashed arrow. Original wild bee and leafhopper images provided by Pexels were modified and used under the Pexels License. The original butterfly image was provided by Katharina Pospisil and subsequently modified.

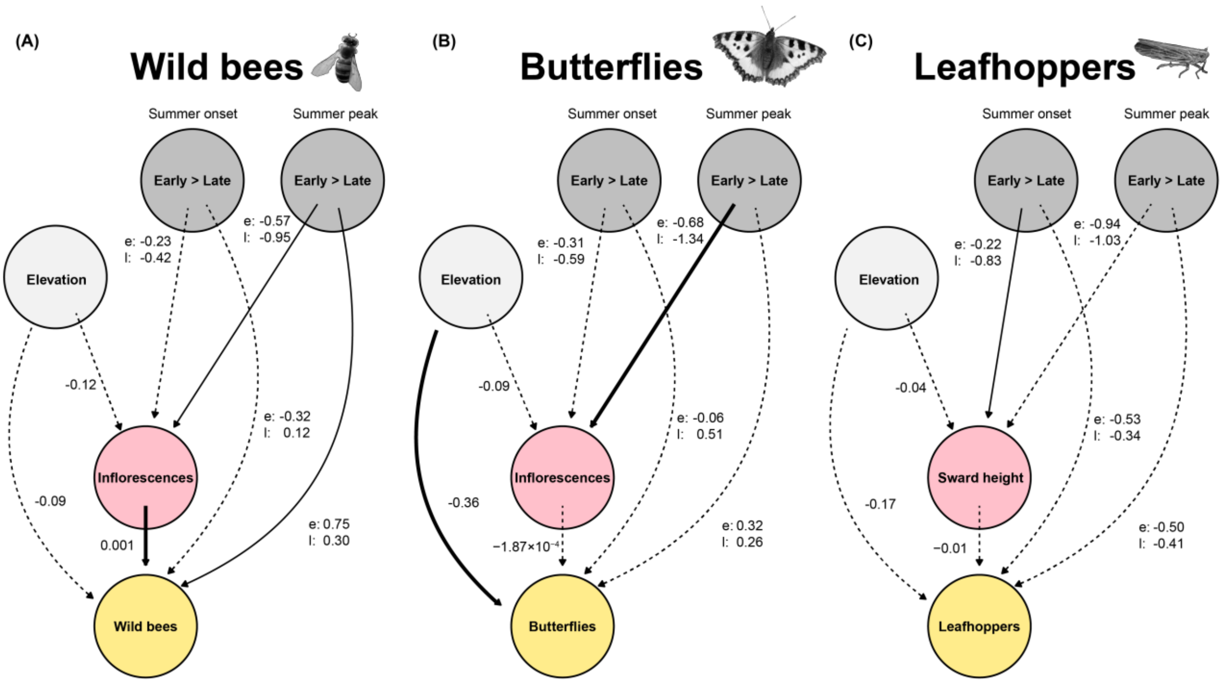

References

Arnold, S. E. J., S. Faruq, V. Savolainen, P. W. McOwan, and L. Chittka. 2010. FReD: The Floral Reflectance Database — A Web Portal for Analyses of Flower Colour. PLoS ONE 5:e14287.

Biedermann, R., and R. Niedringhaus. 2009. The plant- and leafhoppers of Germany: identification key to all species. WABV Fründ, Scheessel.

Ewald, J., T. Köstl, B. Panassiti, and C. Hartung. 2024. Vegetation von Almweiden im Nationalpark Berchtesgaden. In prep.

Gelman, A. 2014. Bayesian data analysis. Third edition. CRC Press, Boca Raton.

Gelman, A., B. Goodrich, J. Gabry, and A. Vehtari. 2019. R-squared for Bayesian Regression Models. The American Statistician 73:307–309.

Higgins, L. G., and N. D. Riley. 1978. Die Tagfalter Europas und Nordwestafrikas. Paul Parey, Singhofen.

Hoffman, M. D., and A. Gelman. 2014. The No-U-Turn Sampler: Adaptively setting path lengths in Hamiltonian Monte Carlo.

Kattge, J., G. Bönisch, S. Díaz, S. Lavorel, I. C. Prentice, P. Leadley, et al. 2020. TRY plant trait database – enhanced coverage and open access. Global Change Biology 26:119–188.

Kudrna, O. 2002. The distribution atlas of European butterflies. Oedippus 20.

Londo, G. 1976. The decimal scale for releves of permanent quadrats. Vegetatio 33:61–64.

Middleton-Welling, J., L. Dapporto, E. García-Barros, M. Wiemers, P. Nowicki, E. Plazio, et al. 2020. A new comprehensive trait database of European and Maghreb butterflies, Papilionoidea. Scientific Data 7:351.

Nickel, H. 2003. The leafhoppers and planthoppers of Germany (Hemiptera, Auchenorrhyncha): patterns and strategies in a highly diverse group of phytophagous insects. Pensoft, Sofia.

Settele, J., R. Feldmann, and R. Reinhardt. 1999. Die Tagfalter Deutschlands. Ulmer, Stuttgart.

Stan Development Team. 2023. RStan: the R interface to Stan.

Vajna, F., J. Kis, and V. Szigeti. 2021. Measuring proboscis length in Lepidoptera: a review. Zoomorphology 140:1–15.

Vehtari, A., A. Gelman, and J. Gabry. 2017. Practical Bayesian model evaluation using leave-one-out cross-validation and WAIC. Statistics and Computing 27:1413–1432.

Westrich, P. 2019. Die Wildbienen Deutschlands. 2., aktualisierte Auflage. Verlag Eugen Ulmer, Stuttgart.

Wiesbauer, H. 2020. Wilde Bienen: Biologie, Lebensraumdynamik und Gefährdung. 2., erweiterte Auflage. Ulmer, Stuttgart (Hohenheim).
